# Technical Study of a Standalone Photovoltaic–Wind Energy Based Hybrid Power Supply Systems for Island Electrification in Malaysia

**DOI:** 10.1371/journal.pone.0130678

**Published:** 2015-06-29

**Authors:** Nahidul Hoque Samrat, Norhafizan Ahmad, Imtiaz Ahmed Choudhury, Zahari Taha

**Affiliations:** 1 Centre for Product Design and Manufacturing (CPDM), Department of Mechanical Engineering, Faculty of Engineering, University of Malaya, 50603, Kuala Lumpur, Malaysia; 2 Innovative Manufacturing, Mechatronics and Sports Laboratory (iMAMS), Faculty of Manufacturing Engineering, University Malaysia Pahang, 26600, Pekan, Pahang, Malaysia; University of California Berkeley, UNITED STATES

## Abstract

Energy is one of the most important factors in the socioeconomic development of a country. In a developing country like Malaysia, the development of islands is mostly related to the availability of electric power. Power generated by renewable energy sources has recently become one of the most promising solutions for the electrification of islands and remote rural areas. But high dependency on weather conditions and the unpredictable nature of these renewable energy sources are the main drawbacks. To overcome this weakness, different green energy sources and power electronic converters need to be integrated with each other. This study presents a battery storage hybrid standalone photovoltaic-wind energy power supply system. In the proposed standalone hybrid system, a DC-DC buck-boost bidirectional converter controller is used to accumulates the surplus hybrid power in the battery bank and supplies this power to the load during the hybrid power shortage by maintaining the constant dc-link voltage. A three-phase voltage source inverter complex vector control scheme is used to control the load side voltage in terms of the voltage amplitude and frequency. Based on the simulation results obtained from MATLAB/Simulink, it has been found that the overall hybrid framework is capable of working under variable weather and load conditions.

## Introduction

Energy was, is and will remain one of the fundamental economic development foundations of any nation. The majority of the island development problems all over Malaysia are mostly related to energy production. Most of the offshore islands in Malaysia use fossil fuels to generate electricity even though Malaysia has a good mix of renewable energy sources such as solar, wind, wave, biomass and hydro. The energy produced by the traditional sources increase greenhouse gas emissions, which may be the key source of global warming. Furthermore, the cost of fossil fuel increases significantly with remoteness. By 2020, it is expected that Malaysia will discharge 285.73 million tons of CO2. This means that CO2 emission will increase by 68.86% compared to the amount of CO2 emitted in 2000. Electric power generation alone contributes 43.40% of the total CO2 released by Malaysia, which is the largest among all sectors [1]. In the previous Kyoto protocol, the Malaysian government signed for a reduction in CO2. For this reason, the government is very concerned about the related environmental issue and wants to reduce the overall CO2 emission. Therefore, island electrification using renewable energy sources in Malaysia is an important way to meet the challenge.

Among the various renewable energy sources, solar energy is the most promising environmental friendly and fastest growing clean and renewable energy source [2]. It has a greater potential than any other power source to solve global energy problems [3]. Malaysia has sunlight throughout the year, so solar energy has huge potential to be exploited, to be a vital source of electric power generation especially for island and remote communities. In Malaysia, the annual average daily solar irradiations are from 4.21 kWh/m^2^ to 5.56 kWh/m^2^. It is estimated that August and November have the highest solar radiation of 6.8 kWh/m^2^ while the lowest is 0.61 kWh/m^2^ in December [4–5]. But the main drawbacks of this system is the power generated by this system is highly dependent on weather conditions. For example, a PV system would not able to generate any power during cloudy periods and at night. In addition, sometimes during a sunny period it intermittently produces power due to the fluctuation of irradiance, which means that the PV system may not be totally satisfy the load demand at each instance. This problem can be solved by integrating the PV system with energy storage elements and/or other green energy source (such as ultra-capacitor bank wind, battery bank, wind, ocean wave, fuel cell and hydrogen storage tank) in a suitable hybrid framework. Cetin et al. [6] established a PV–wind-fuel cell hybrid energy system for residential application at the Clean Energy House in Denizli, Turkey. Valenciaga et al. [7] described a hybrid PV-wind system with supervisory control. In this reference paper, wind energy system used as the main power generation source whiles the solar energy system would play a complementary role. In this case, the only AC load is considered and supplied by the hybrid system. Onar et al. [8] designed and modeled a wind-fuel cell-ultracapacitor based hybrid system for grid-independent applications. They developed a detailed dynamic simulation model which allows designing and analyzing any wind-fuel cell-ultracapacitor hybrid system with various parameters and power levels. The major contribution of this research work is the renewable sources hybridization with fuel cell systems using short and long-term storage strategies with proper hybrid power controllers. Uzunoglua et al. [9] developed a PV-fuel cell-ultracapacitor hybrid systems for sustainable power generation. In the proposed system, the PV system during adequate solar irradiation activated the electrolyzer to produce hydrogen for future use and transfers energy to the load side if possible. Whenever the PV system unable meets load demands for inadequate solar irradiation, the fuel cell provides power by using the stored hydrogen to meet the remaining load. The ultracapacitor bank meets the load demand if the load demand rate increases the outside limits of fuel cell. With reference to the related literature [3–4, 10], wind energy conversion could be seriously considered for generating electric power in Malaysia. In Malaysia, the speed of the wind is in the range of 2 m/s to 13 m/s and it varies from season to season [10]. In this region, the strongest wind comes from the South China Sea to the East Coast in the northeast monsoon (from September to March). So wind energy can be considered as one of the environmental friendly hybrid power generating sources for island electrification in Malaysia. Also, in this study battery used as energy storage device because of its quick response, mature technology, high efficiency and low cost [11–14].

Among them, Delfino et al. [15] presented a grid integrated wind turbine- fuel cell system. Khan et al. [16–17] described a small wind-fuel cell hybrid model and analyzed its life cycle. Onar et al. [18] investigated detailed dynamic modeling, design, control and simulations of wind/solar/fuel cell/ultra-capacitor hybrid system. A wind, PV and wave based large-scale hybrid system integration and grid connection has been reported in Lund [19]. Bhende et al. [20] presented a stand-alone wind- fuel cell based supply system. In De Battista et al. [21]power conditioning for a wind-hydrogen storage based system was analyzed and discussed. Besides these, some others works related with the renewable integration and related energy storage are briefly discussed in references [22–25]. In all the references [6–9, 15–21],some authors discussed energy storage devices for a hybrid system, some discussed grid integration of a hybrid system, sizing and cost optimization. But they are silent about the PV-Wind energy based hybrid system design for island communities.

This study focuses on developing a complete dynamic simulation model to design, control and analyze the overall system performance of a feasible PV-wind hybrid renewable power generation system for residential use. This simulation model can be used not only for investigating the PV-Wind hybrid system performance, but also for sizing and designing the HRES to meet the system load demands under any available meteorological condition. Using a DBBC, a control algorithm is developed between the dc-link and battery bank to maintain the constant dc-link voltage, and a switch mode inverter with complex vector control scheme is placed at the load side end to control the load side voltage. In addition, a simple passive L-C filter is placed after the inverter at the load side end to eliminate the unwanted high frequency harmonics, which are generated by the VSI based on the inverter switching frequency. The proposed battery storage PV-Wind stand-alone hybrid system in this paper has been modeled, controlled and simulated using Matlab, Simulink and Simpower system software packages. In addition, results obtained from simulation are described to verify the effectiveness of the proposed HRES under variable meteorological conditions.

The sequential workflow hints of this paper are as follows: the complete mathematical modeling of PV-Wind HRES has been presented with the necessary equations shown in Section 2. In addition, Sections 2 describes the dc-link voltage control algorithm and complex vector control scheme for load side VSI. In Section 3, the Perhentian Islands is considered as a potential area for generating electric power from PV and wind energy sources based on the meteorological data. Section 4 presents all the necessary simulation results and discussions to check the feasibility of the HRES. Finally, in Section 5, a conclusion is drawn by combining all the key points of the study.

## System Description

**Fig 1:**
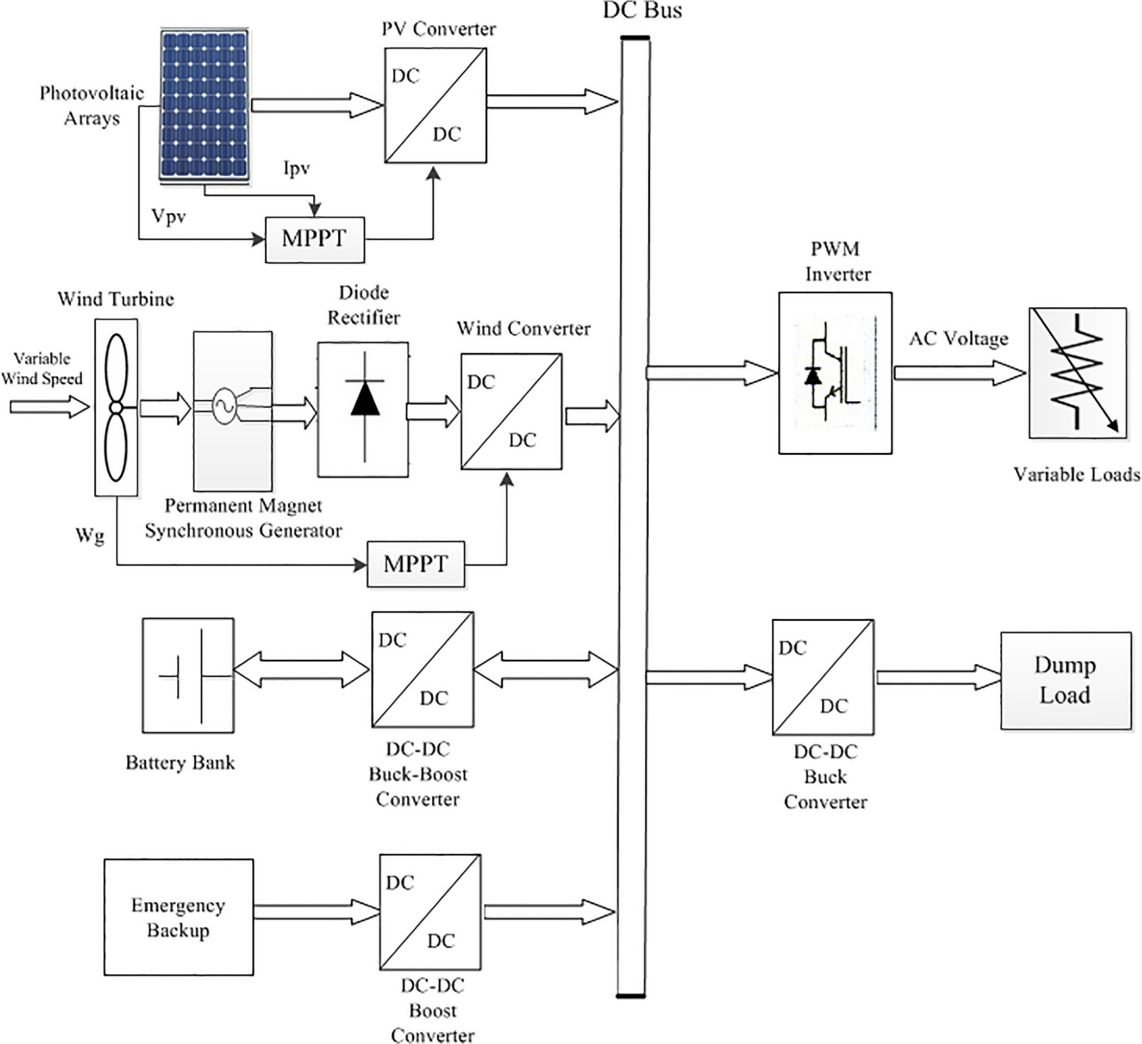
Block diagram of the proposed PV-Wind hybrid system.

In this section, the detailed dynamic simulation model is briefly described for a PV-Wind hybrid renewable power generation system. The proposed hybrid system consists of a PV system, a wind energy system, a battery bank, a DBBC with proportional integral (PI) control duty cycle and a pulse width modulation (PWM) VSI located at the load side end. The solar PV system consists of a PV array and a DC-DC converter with a maximum power point tracking (MPPT) algorithm. The wind energy system is configured by a wind turbine with a permanent magnet synchronous generator (PMSG), a DC-DC converter with MPPT and an AC-DC three-phase uncontrolled rectifier. Fig 1 shows the complete block diagram of the stand-alone PV-Wind HRES.

### PV system modeling and characteristics

**Fig 2:**
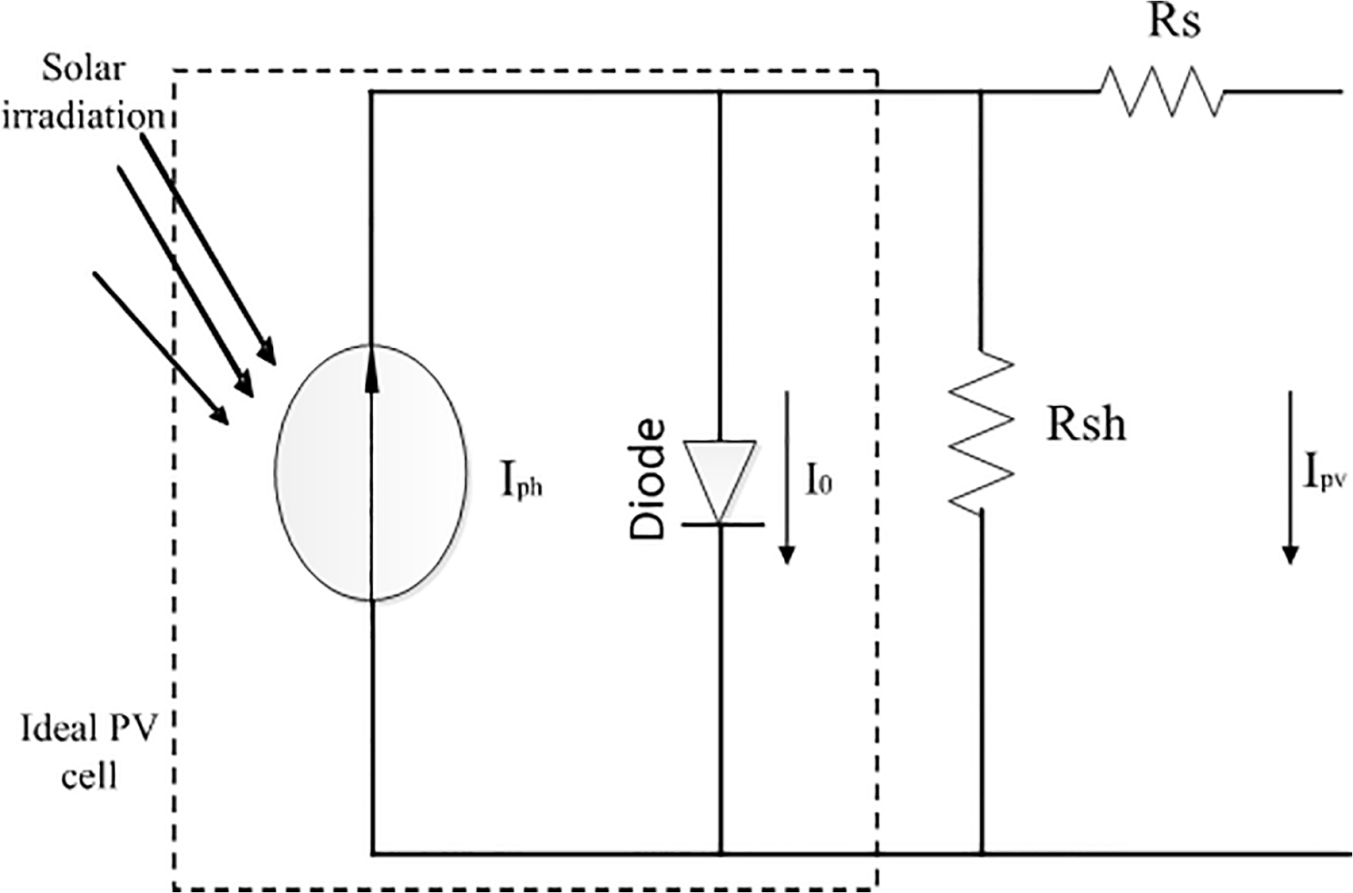
Equivalent circuit diagram of a single-diode PV model.

The solar PV system is a method of generating electrical energy by converting solar photon energy into direct current electricity using a solar cell or PV cell. The PV cell is the smallest unit of the solar PV system and each PV cell produces around 0.5V. Cells are further connected in series or/and parallel combination to form a PV array. Different PV cell configurations can be used to illustrate the different V-I characteristic curves such as single diode model, two diode model, and Rs-Rp model. But due to the degree of accuracy and simplicity, the single diode model has been used in some previous works. For this reason, the PV cell single diode configuration has been selected for this study. Fig 2 presents the widely used one-diode equivalent circuit model of PV cell [26]. PV model V-I characteristics can be expressed using some nonlinear mathematical exponential equations. The ideal relationship between PV voltage and current can be expressed as follows [26–27]:

$$I_{PV}=I_{ph}-I_{D}-I_{sh}=I_{ph}-I_{0}[\text{exp}\frac{q\times (V_{PV}+I_{PV}R_{s})}{A_{q}K_{b}T}-1]-\frac{V_{PV}+I_{PV}R_{s}}{R_{sh}}$$ (1)

where *I*_*PV*_ is the output current of the PV cell (A), *I*_*ph*_ the photocurrent, *I*_*D*_ the diode current, *I*_*sh*_ the current through the shunt resistance, *I*_0_ the reverse saturation current, K the Boltzmann constant = 1.38×10^−23^ (*J* / *K*), q the charge of electron = 1.6×10^−19^ (*C*), T the cell temperature (K), *V*_*PV*_ the output terminal voltage of the PV cell (V), A the quality factor (lies between 1.2–1.6 for crystalline silicon), *R*_*s*_ the series resistance (Ω) and *R*_*sh*_ the shunt resistance (Ω).

**Fig 3:**
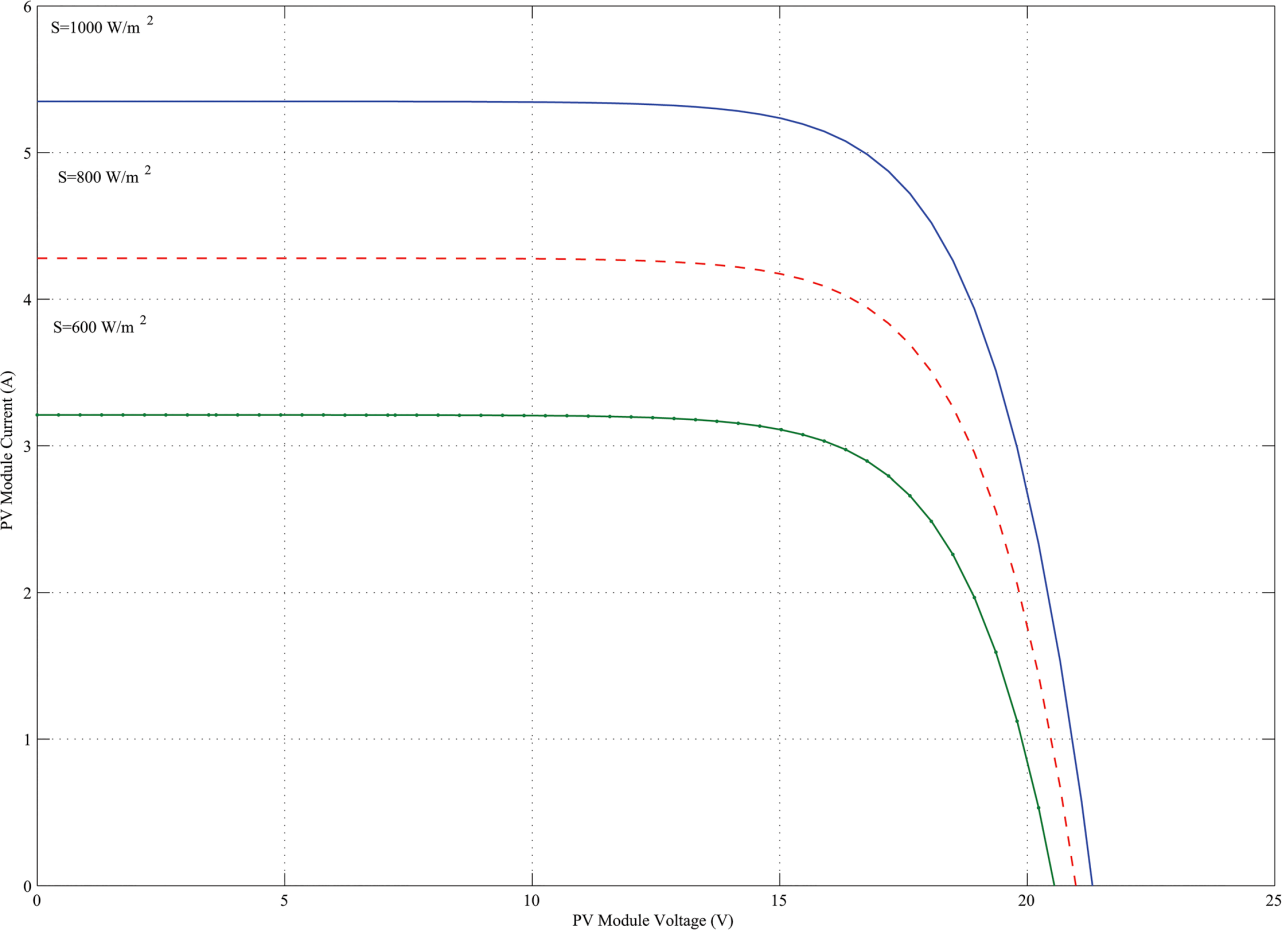
I-V characteristics of KOYCERA KC85T-87W PV model with varying irradiation.

**Fig 4:**
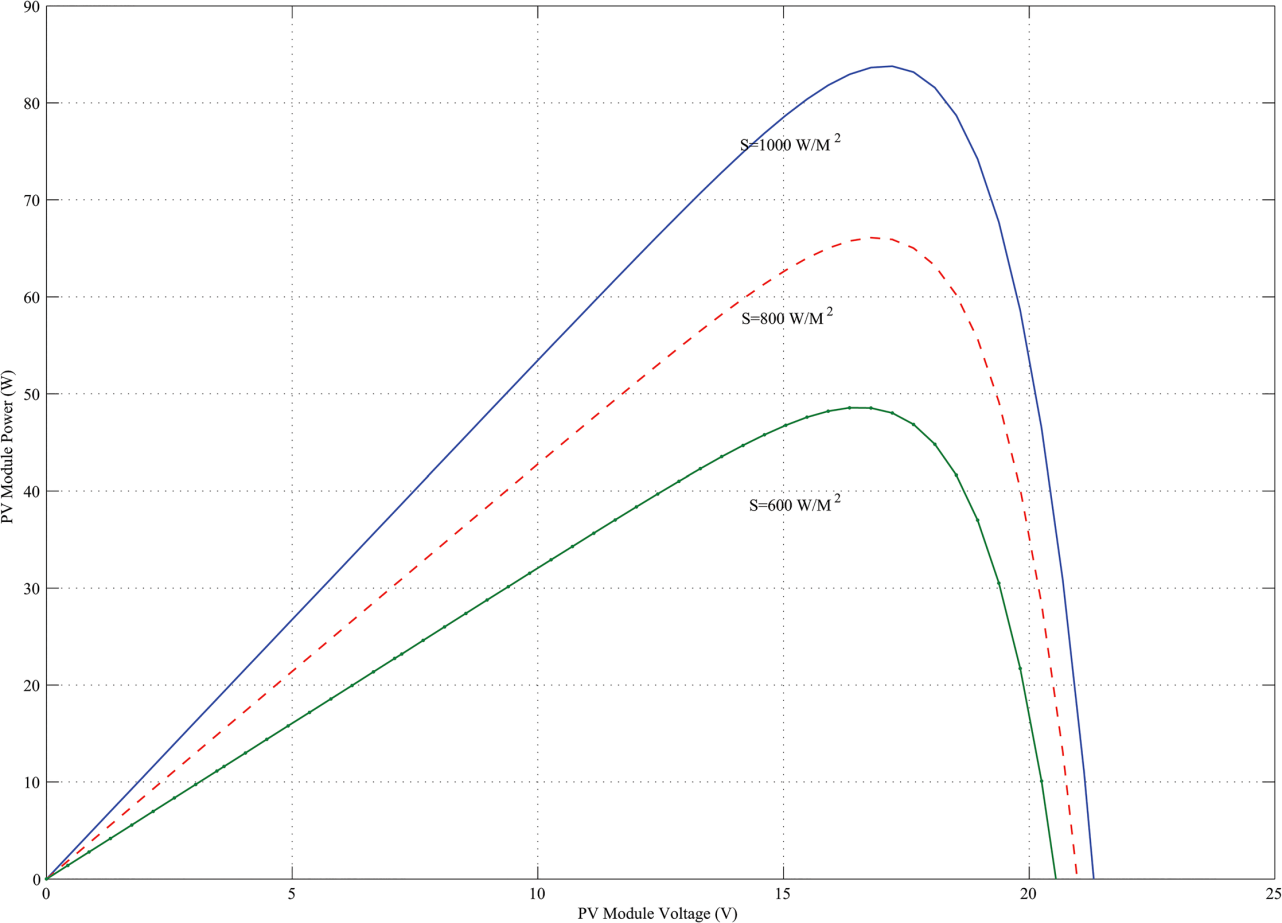
P-V characteristics of KOYCERA KC85T-87W PV model with varying irradiation.

The output power from the PV array is given by

$$P_{PV}=V_{PV}I_{PV}\eta_{conv}$$ (2)

where *η*_*conv*_ is the efficiency of the DC-DC converter (typically 90–95%). In this paper, for a 400W power generation, total five KOYCERA KC85T-87W PV model is used and all the models are connected in series in combination with one another. The KOYCERA KC85T-87W PV model P-V and I-V characteristics are obtained according to the value of the variables *I*_*ph*_, *I*_0_, *R*_*sh*_ and *R*_*s*_. The value of the variables can be obtained from [26]; they usually provide values for open circuit *V*_*PV*_ and *I*_*PV*_, short circuit and maximum power point and finally the number of PV cells. The I-V and P-V characteristics of a PV module operating at 25°C standard temperature and different solar irradiance are shown in Figs 3 and 4.

**Fig 5:**
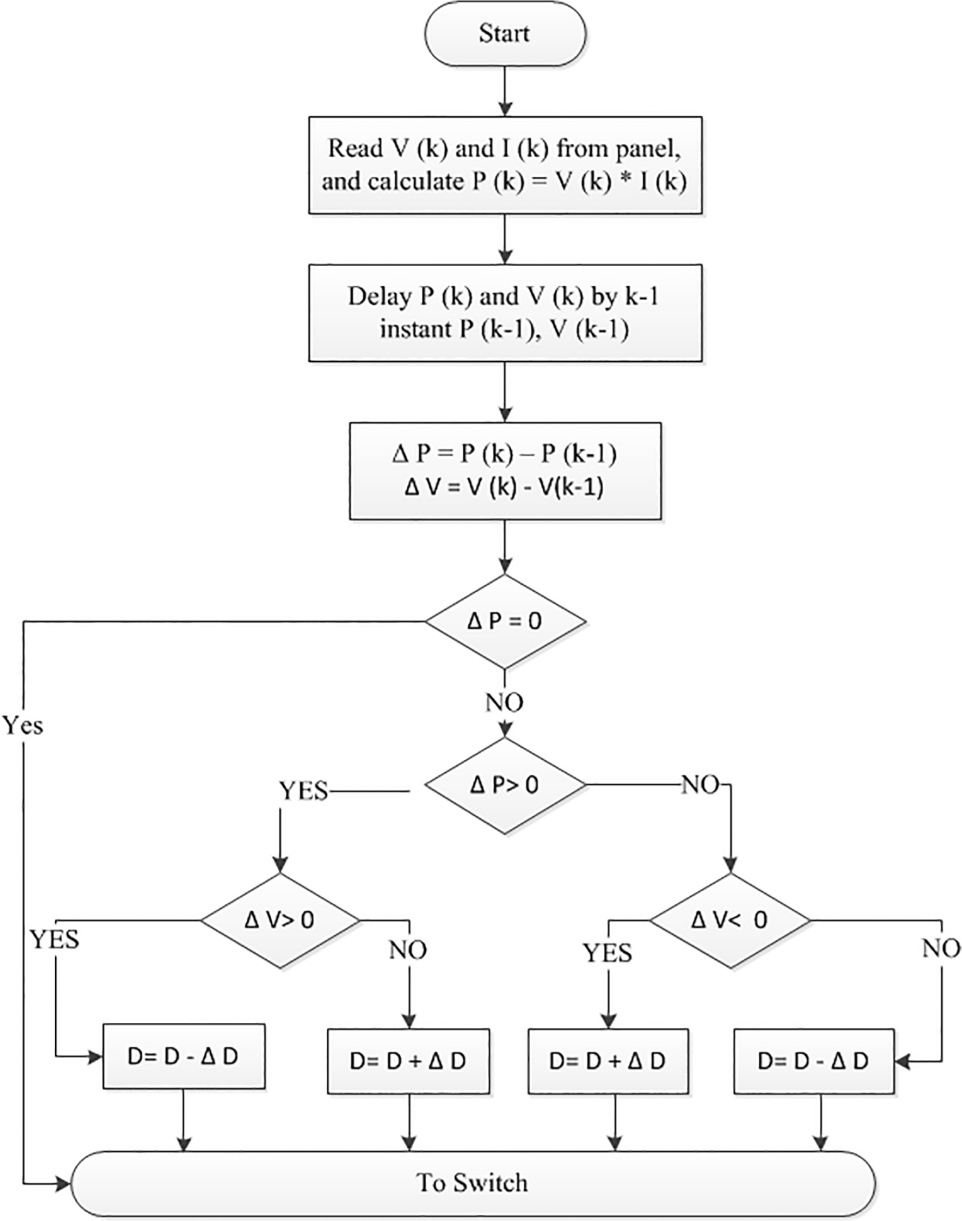
The flowchart of the P&O Algorithm.

**Fig 6:**
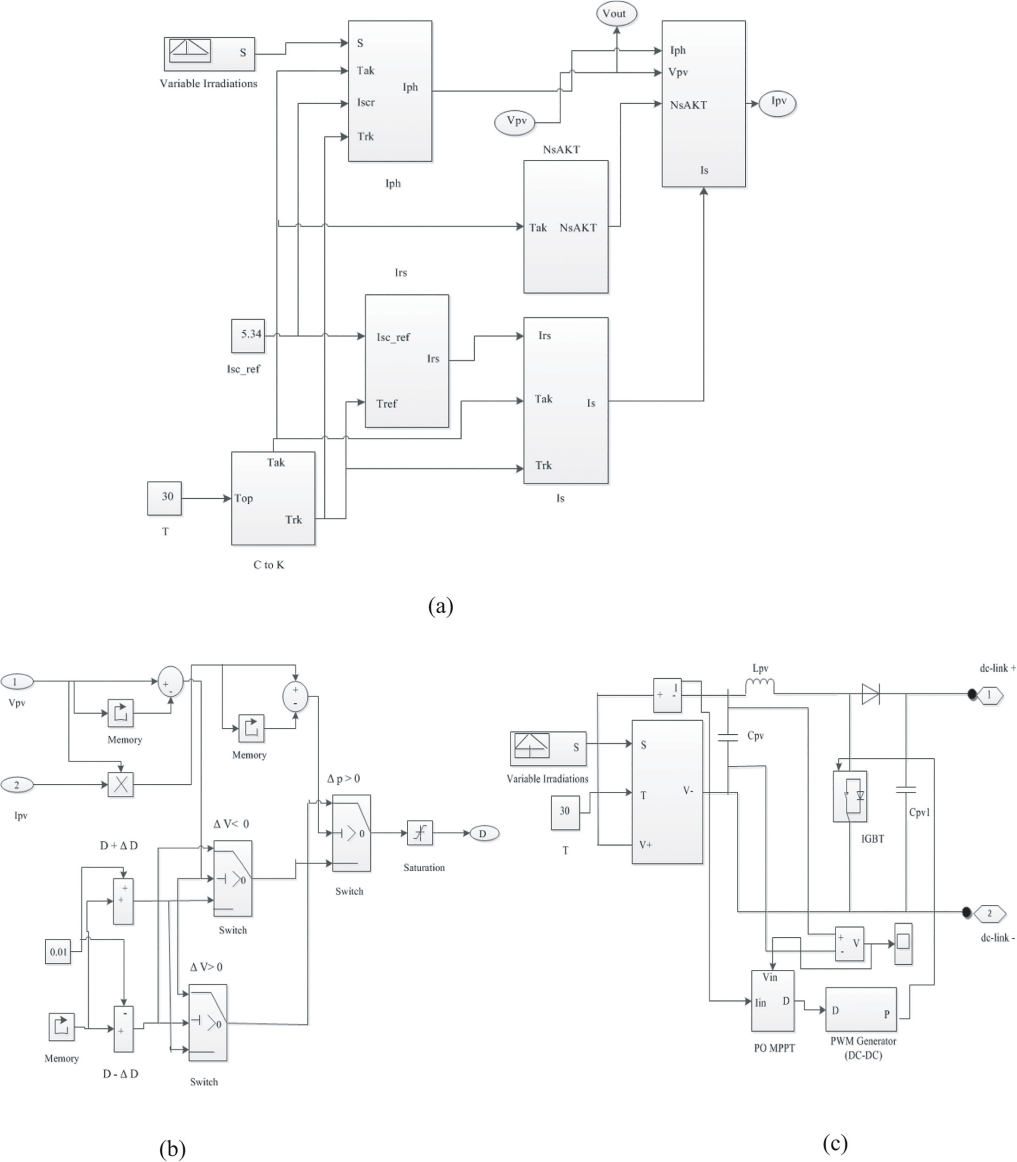
The PV model Simulink diagram with MPPT. (A) Simulink model of PV. (B) MPPT model. (C) Complete Simulink model of PV with MPPT.

The maximum output power of the PV module varies according to load current or solar irradiation. Therefore, a proper control strategy is needed to use the PV model more efficiently as an electric power source by developing a MPPT. There are many different types of MPPT algorithm discussed in [26–28].Due to the simplicity and the smaller number of measured variables, the perturbation and observation technique (P&O) is the most widely used among them. In P&O algorithm, the last perturbation sign and the last increment sign in the power of the solar module are used to select what should be the next perturbation. A slight (ΔD = 0.01) perturbation is introduced in the system. If there is an increment in the power, then the perturbation is continued (D + ΔD) in that direction and if the power decreases, the perturbation should be reverses (D − ΔD). The flow chart of the P&O MPPT algorithm is shown in Fig 5 [22]. According to Hasan and Natarajan [26–27], Eqs (1) and Eqs (2); the PV model with MPPT is developed using MATLAB/Simulink, which is depicted in Fig 6 (A), 6(B) and 6 (C).

### Wind turbine modeling

Wind turbine extracts the kinetic energy from wind and then converts it into mechanical energy that is fed into the electrical generator through a shaft to generate electricity. The mechanical power developed by a wind turbine [29–32] can be formulated as

$$P_{m}=\frac{1}{2}\rho A_{t}C_{p}\;(\lambda ,\beta )\times V_{w}{}^{3}=\frac{1}{2}\rho A_{t}C_{p}\times {\left(\frac{\omega_{m}R}{\lambda }\right)}^{3}$$ (3)

where *P*_*m*_ is the mechanical power (W), *C*_*p*_ is the power coefficient, *λ* the tip speed ratio, A is the wind turbine swept area (*m* ^2^), V_w_ is the wind speed (*ms* ^−1^), *ρ* is the density of air *kg* / *m* ^3^, *ω*_*m*_ is the turbine rotor angular speed in *rads* ^−1^ and R the wind turbine rotor radius (m). The output power is determined by the Cp of the wind turbine if the swept area and wind speed are assumed to be constant.

The relationship between the tip speed ratio and the angular rotor speed can be expressed as

$$\lambda =\frac{\omega_{m}R}{V_{w}}$$ (4)

Maximum power from the wind turbine can be extracted if the turbine operates at maximum Cp (Cp-opt). Therefore, it is essential to keep the turbine rotor speed at the optimum value of tip-speed ratio (λ_opt_) with variation of wind speed. Using Eqs (3) and (4), the target optimum power of the wind turbine can be expressed as [31–32]:

$$P_{m}=\frac{1}{2}\rho AC_{p\_opt}\times {\left(\frac{\omega_{m\_opt}R}{\lambda_{opt}}\right)}^{3}=K_{opt}\times {\left(\omega_{m\_opt}\right)}^{3}$$ (5)

Where

$$K_{opt}=\frac{1}{2}\rho AC_{p\_opt}{\left(\frac{R}{\lambda_{opt}}\right)}^{3}$$ (6)

$$\omega_{m\_opt}=\frac{\lambda_{opt}}{R}V_{\;w}=K_{w}V_{w}$$ (7)

Therefore, the optimum torque can be given by [29–32]

$$T_{m\_opt}=K_{opt}{\left(\omega_{m\_opt}\right)}^{2}$$ (8)

**Fig 7:**
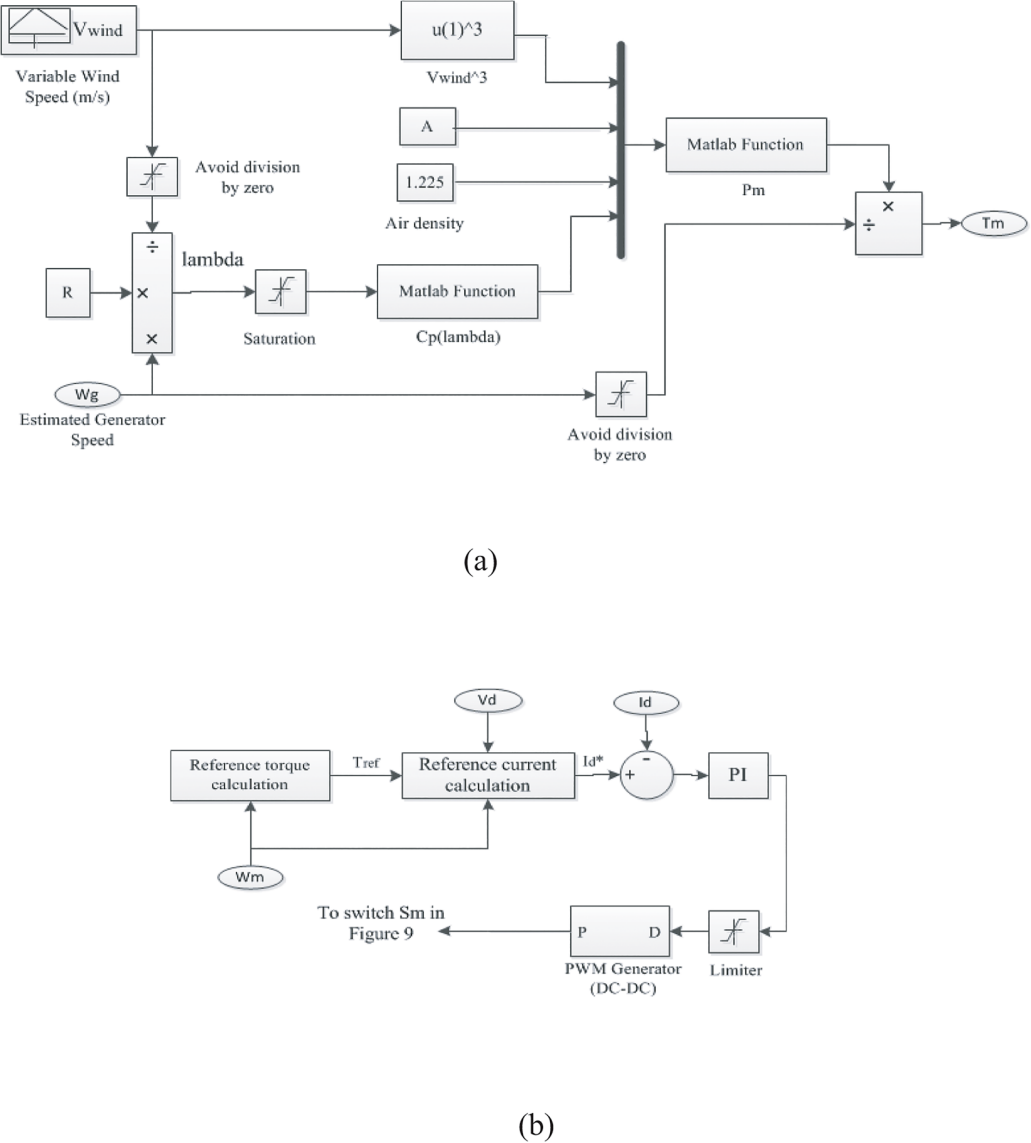
Wind turbine Simulink diagram with MPPT. (A) Wind turbine simulink model; (B) MPPT model.

From the above equation and related literature [31–32], it can be observed that the maximum power extraction from a wind turbine is a function of the turbine rotor speed at different wind speeds. In this paper, according to the Eqs (3)–(8) a maximum power extraction algorithm is developed [31–32]; a wind turbine model with MPPT is developed using MATLAB/Simulink, which is illustrated in Fig 7(A) and 7(B).

### Modeling of battery

The electric utility system reliability and efficiency improved by the energy storage devices. Among the various energy storage devices, battery is the most common storage device used for the electrical energy storing. Recently there are many battery models are developed in references [33–36]. In this paper, a typical battery model [36] is implemented. This model uses only the state of charge (SOC) of the battery as a state variable to avoid the algebraic loop problem. In addition, the model developed [36] can precisely characterize four types of battery chemistry including lead-acid battery.

**Fig 8:**
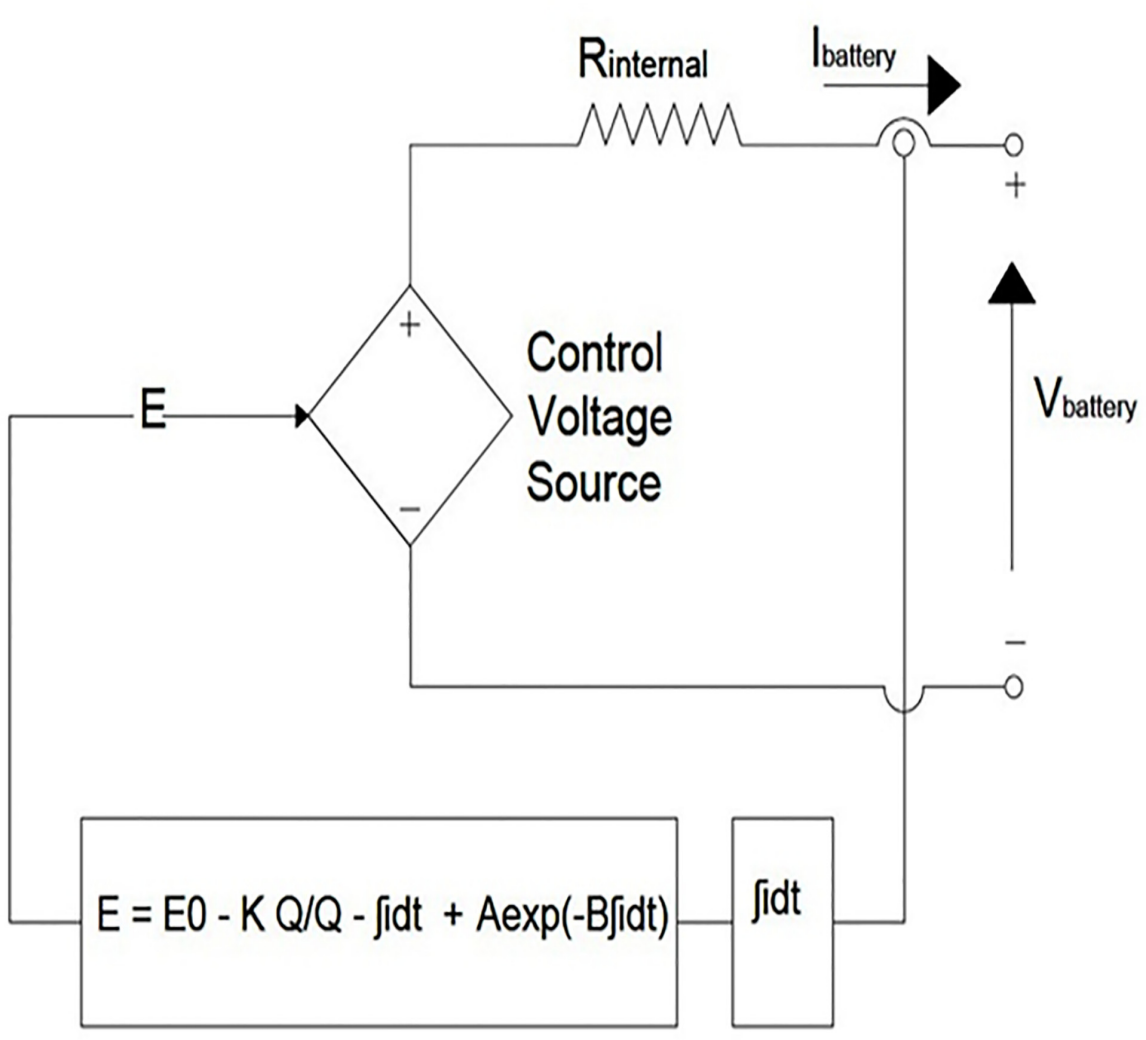
Non-linear typical battery model [36].

In this paper, the battery is modeled using a controlled series connected voltage source with a constant resistive value, shown in Fig 8, where the controlled voltage source is expressed by the following equation [36]:

$$E=E_{0}-K\frac{Q}{Q-\int \;idt}+A\;\text{exp}(-B\int \;idt)$$ (9)

$$V_{Battery}=E-R_{in}I_{Battery}$$ (10)

where E_0_ is the no load voltage of the battery (V), Q is the battery capacity (Ah), K is the polarization voltage (V), A is the exponential zone amplitude (V), V_Battery_ is the battery voltage (V), B is the exponential zone time constant inverse (Ah)^-1^, R_in_ is the internal battery resistance (Ω), ∫idt is the charge drawn and supplied by the battery (Ah) and I_Battery_ is the battery current (A)

**Fig 9:**
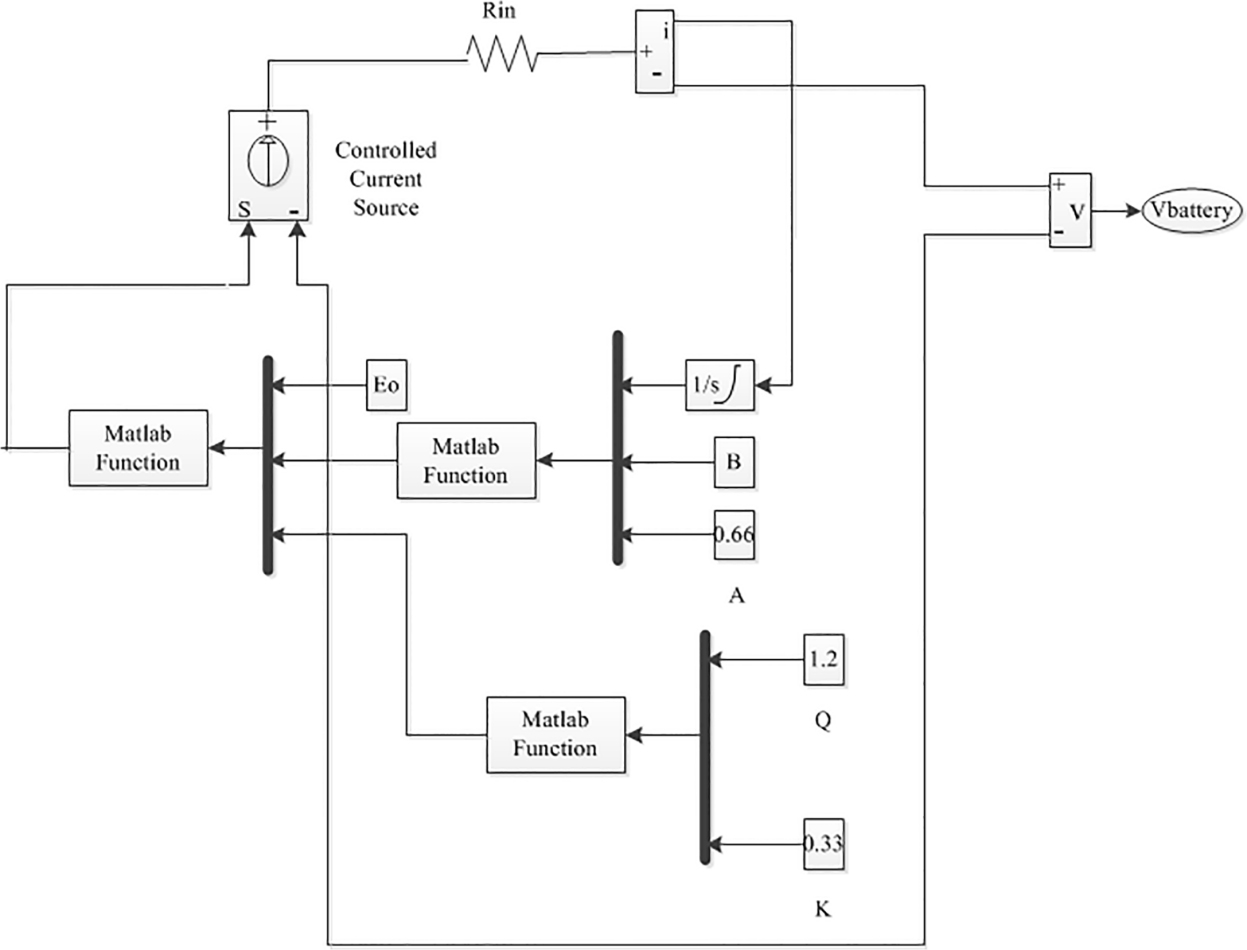
The battery model Simulink diagram.

Based on Eq (9), the typical battery model is developed in MATLAB/Simulink and connected to a DBBC using controlled voltage source, shown in Fig 9.

### DC-link voltage control

**Fig 10:**
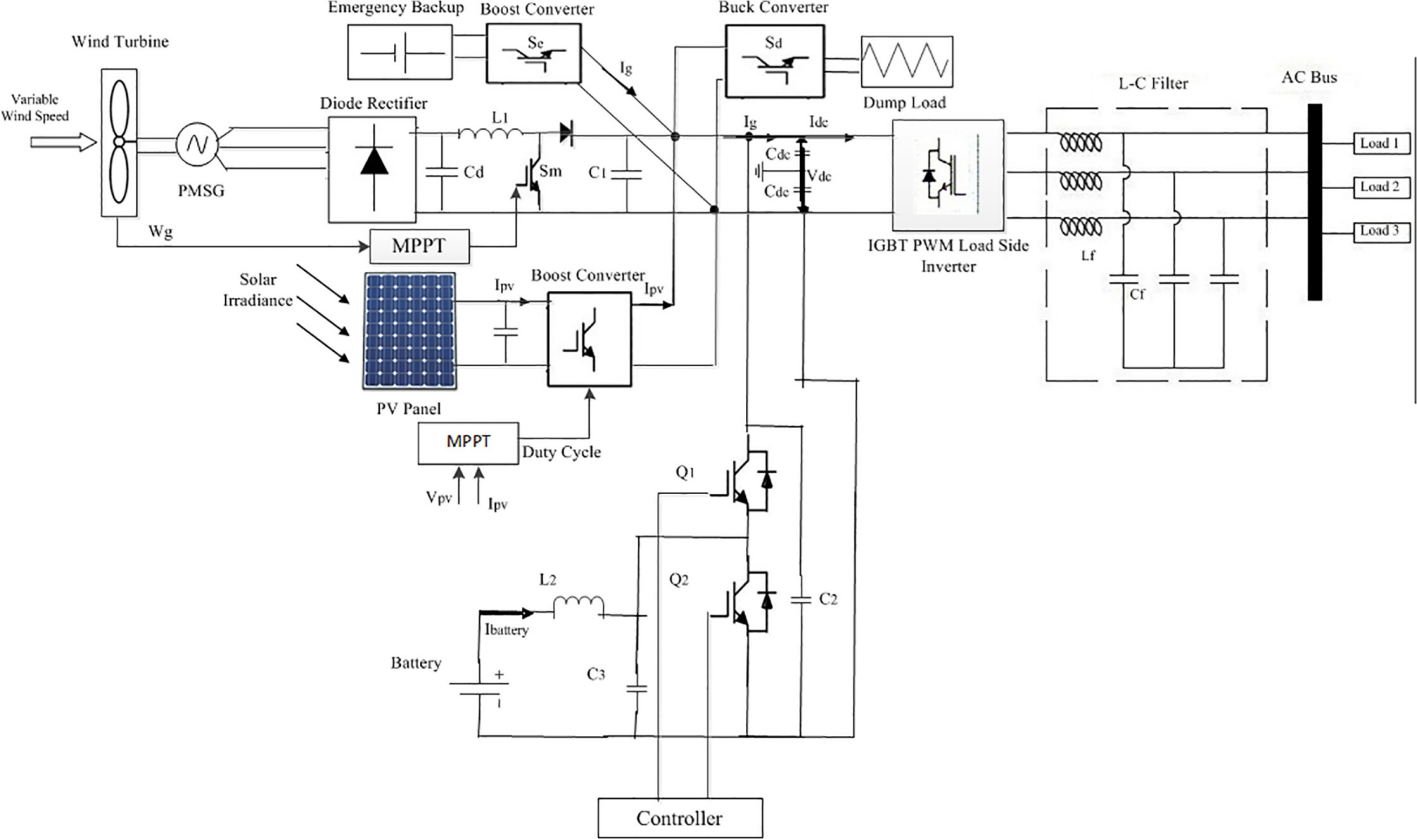
The proposed stand-alone PV-Wind hybrid system circuit topology with dump load and emergency backup.

The proposed stand-alone PV-wind hybrid power generation system circuit topology with dump load and emergency backup is depicted in Fig 10. In this proposed system, a neutral wire is inserted between the dc-link capacitors which are connected before the VSI for feeding the single phase load as well as the three phase loads, shown in Fig 10.

**Fig 11:**
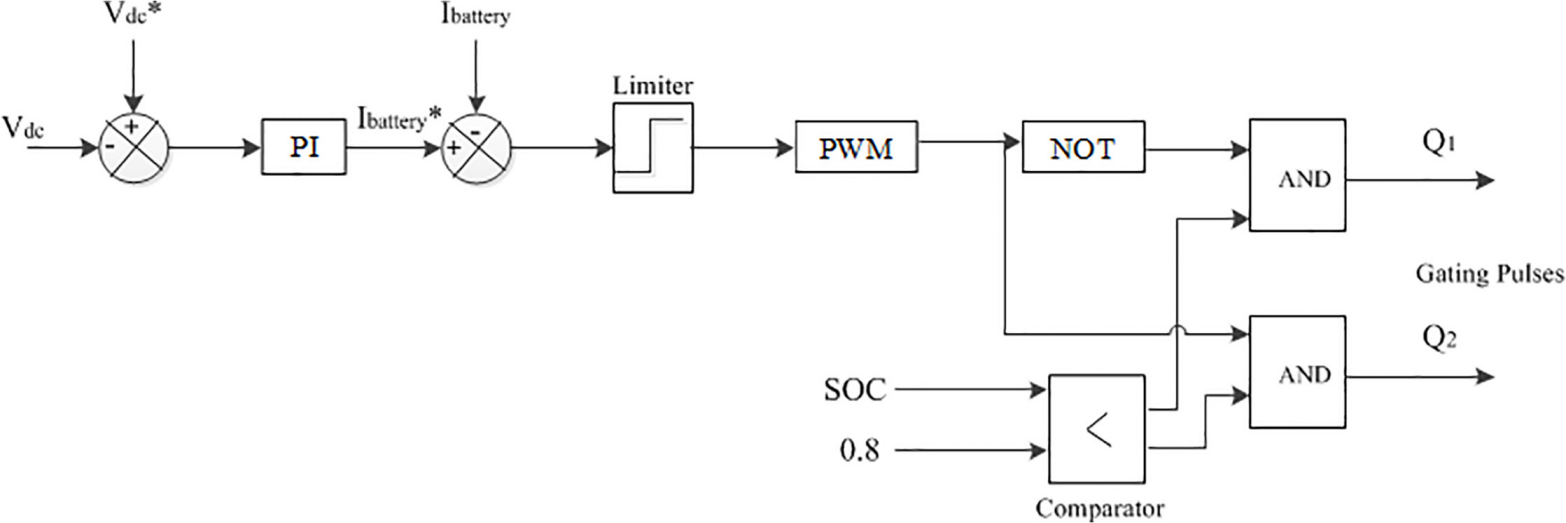
The schematic diagram of DBBC converter controller.

In this paper, the dc-link side is connected to a battery bank through DBBC; the main objective of the DBBC control is to maintain a constant dc-link voltage as a reference value and discharge/charge current from/to the battery bank according to the required load power. The battery bank DBBC controller schematic diagram shown is Fig 11. The battery bank voltage can be kept lower as compared to the dc-link reference voltage (V_dc_*) by using DBBC and so a smaller number of battery are required to be connected in series. In the proposed system, the battery bank voltage is kept at around 300 V whereas V_dc_* = 650 V. In this study, the depth of discharge of the battery bank is considered to be 60% [20].In addition, it is assumed that it should provide electric power to the 2.5 kW load for approximately an hour when the generated hybrid power is zero. The detailed calculation of the battery bank rating is discussed in S1 Appendix.

In Fig 10, the value of the switching frequency, inductor and capacitors used in DBBC is important for its conduction mode operation of it. The inductor used in the battery bank side to show the lower ripple current results, which increases the lifetime and efficiency of the battery bank. Conduction mode operation of DBBC also depends on input and output current. The value of the inductor and capacitors are as follows [20]:

$$\text{Inductance}\;L_{2}=\frac{V_{Battery}\times (V_{dclink}-V_{Battery}\;)}{I_{Battery}\times f_{s}\times V_{dclink}}$$ (11)

$$\text{Buck mode capacitance}\;C_{2}=\frac{k_{L}\times I_{Battery}}{8\times f_{s}\times V_{Battery\;(ripple)}}$$ (12)

and

$$\text{Boost mode capacitance}\;C_{3}=\frac{D_{Boost}\times I_{dclink}}{f_{s}\times V_{dclink\;(ripple)}}$$ (13)

where V_Battery_ is the voltage of the battery bank, I_dclink_ is the dc-link current, V_dclink_ is the dc-link voltage, D is the duty cycle. V_dclink(ripple)_ is the boost side output desired ripple voltage, V_Battery(ripple)_ is the buck side output desired ripple voltage, f_s_ is the switching frequency, k_L_ is the estimated coefficient of indicator ripple current at buck side and I_Battery_ is the battery bank current.

**Fig 12:**
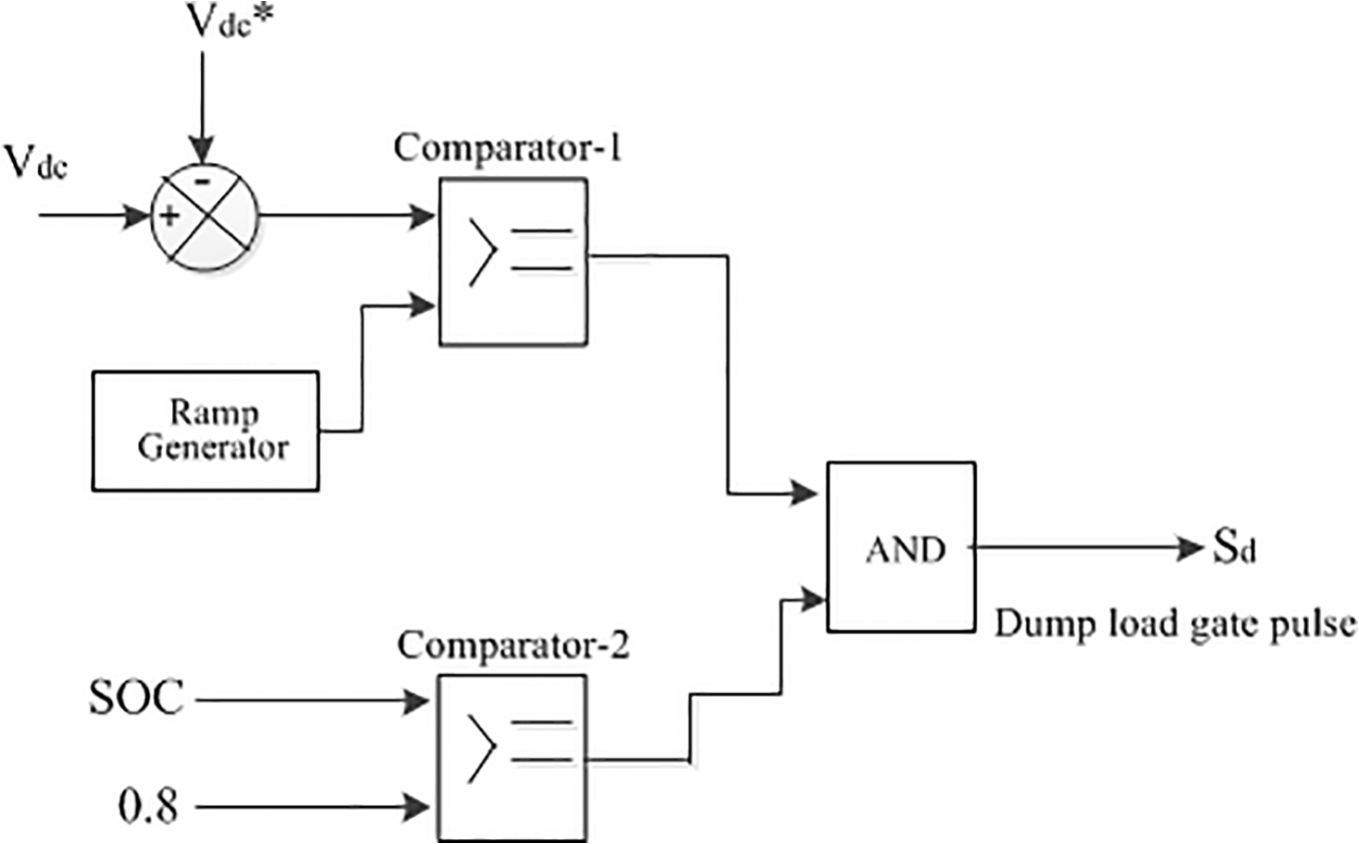
The schematic diagram of dump load controller.

The battery bank in this paper can act either as a sink or as a power supply. As a result, due to the weather conditions, it should charge or discharge within specified limits when there is surplus or a lack of hybrid power. In this study, the surplus power due to the high solar and wind power condition at first supply power to the battery bank until it reaches its upper limit charge carrying capacity and after that the dump load absorbs the additional power. The dump load controlled via the chopper control is shown in Fig 12. In this case, the controller switching decision is made by comparing the present status and upper limit of SOC.

**Fig 13:**
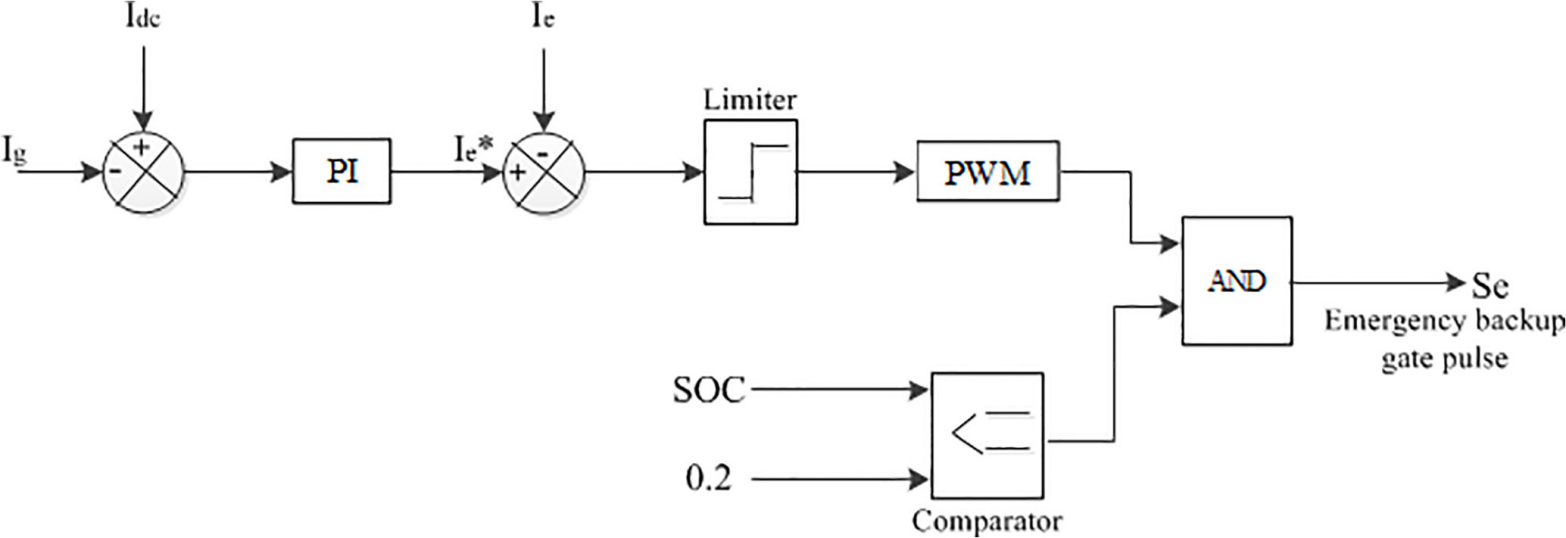
The schematic diagram of emergency backup controller.

**Fig 14:**
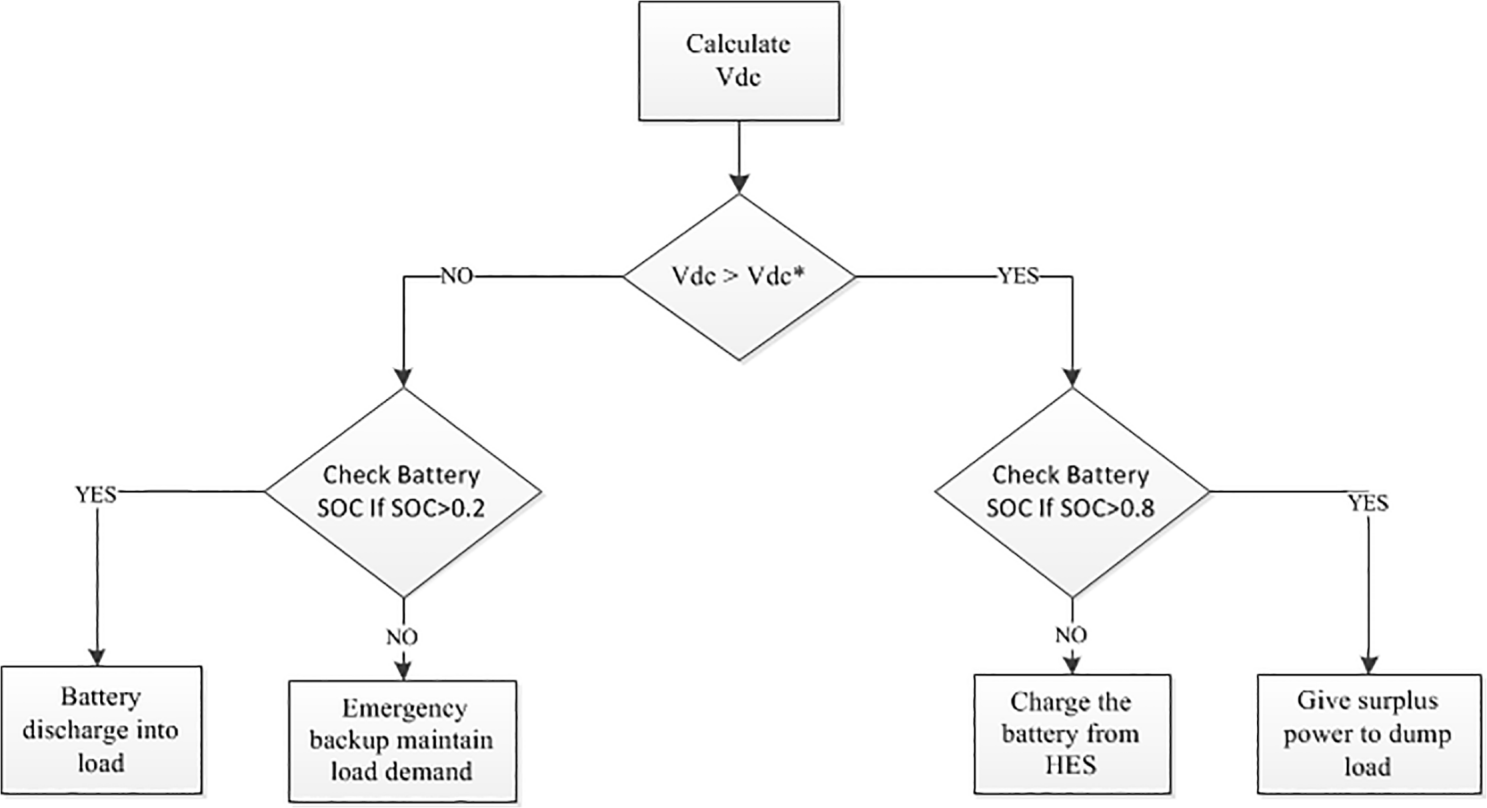
Dc-link voltage control algorithm.

The battery bank may not able to meet the system load demand at each instance in case of long term no or lower PV and/or wind power condition. For this reason, an emergency backup is integrated with the proposed hybrid system in this paper. The control algorithm of the emergency backup is shown in Fig 13. A flow chart is depicted in Fig 14 based on the above control coordination among the different sources, battery, emergency backup and dump load, where the lower and upper limit SOC the of the battery bank is kept at 0.2 and 0.8, respectively [20].

### Load side VSI control

A three phase space vector control VSI is used at the load end as an interface element between the dc-link voltage and the system load. It is used to control the voltage and frequency at the system load end. In this study, the system load voltages should be controlled in terms of voltage amplitude and frequency because there is no power grid connection. The space vector control method is used to control the system output voltage during the variation in required hybrid power or load power.

Based on the synchronously rotating frame described in [31–32], the space vector control technique is used in this proposed system. The three-phase I_a,_, I_b_, I_c_ currents and V_a_, V_b_, V_c_ voltages should be measured and transformed from the stationary reference a-b-c frame to the rotating reference d-q frame using the preferred output load voltage frequency. In this paper, the specified output phase voltage root-mean-square (RMS) value and the frequency are 220 V and 50 HZ, respectively.

The equations of voltage using reference rotating d-q frame transformation are taken from the [32]:

$$v_{d}=v_{di}-L_{f}\frac{di_{d}}{dt}+L_{f}\omega i_{q}$$ (14)

$$v_{q}=v_{qi}-L_{f}\frac{di_{q}}{dt}-L_{f}\omega i_{d}$$ (15)

By using the d-q reference rotating frame transformation, the active and reactive power is given by:

$$\text{Active power}\;P=\frac{3}{2}(v_{d}i_{d}+v_{q}i_{q})$$ (16)

$$\text{Reactive Power}\;Q=\frac{3}{2}(v_{d}i_{q}+v_{q}i_{d})$$ (17)

The active and reactive power equations will be as follows if the reference rotating frame is as v_q_ = 0 and v_d_ = │V│:

$$P=\frac{3}{2}v_{d}i_{d}=\frac{3}{2}\left|V\right|i_{d}$$ (18)

$$Q=\frac{3}{2}v_{d}i_{q}=\frac{3}{2}\left|V\right|i_{q}$$ (19)

**Fig 15:**
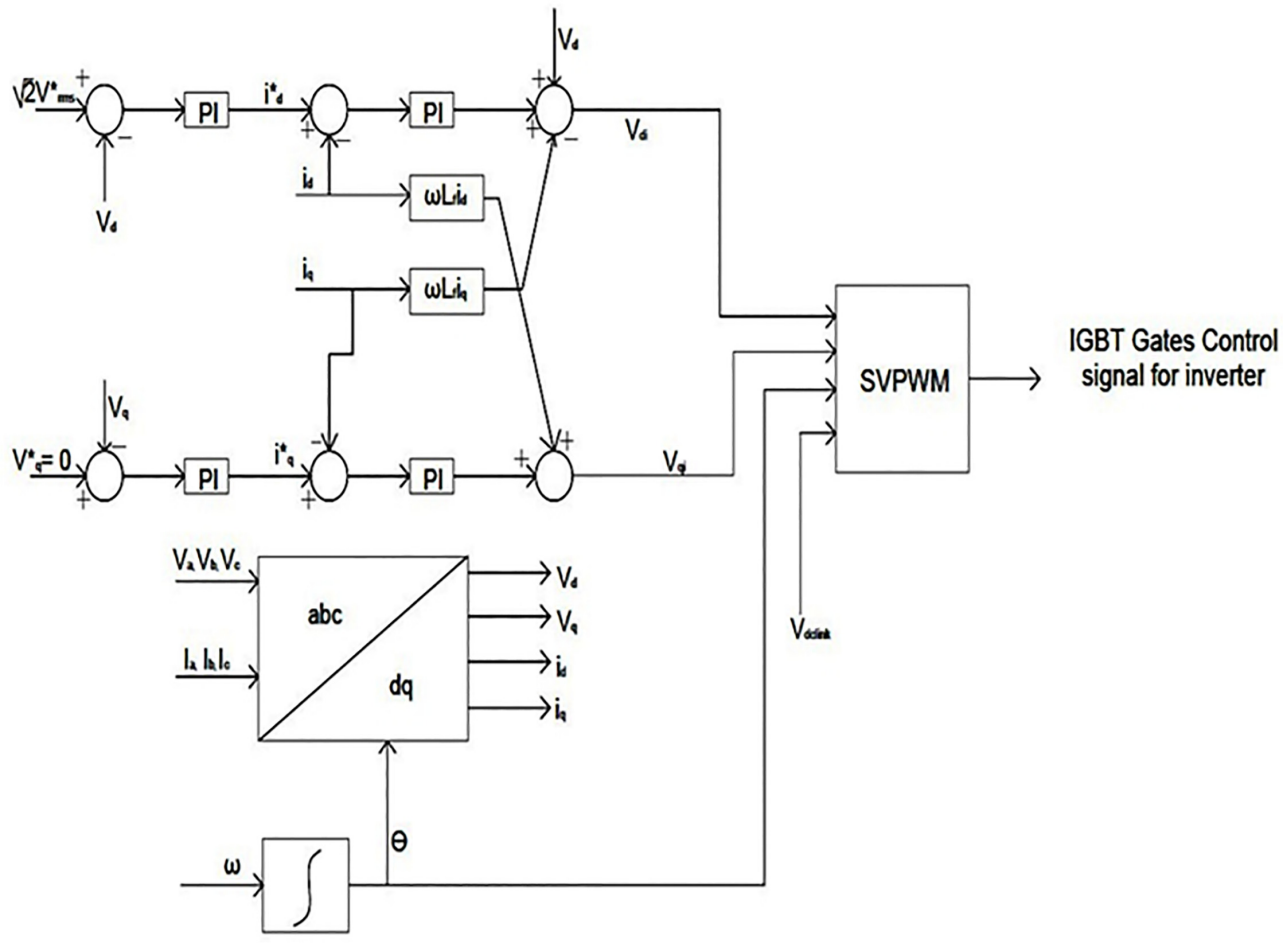
The three-phase load side VSI controller.

Therefore, the active and reactive power can be controlled by controlling direct and quadreature current elements, respectively. And for the resistive load case V_d_* can be expressed as:

$$V_{d}^{*}=\sqrt{2}V_{RMS}^{*}$$ (20)

where V*_RMS_ is the RMS reference value of the output phase voltage. In this control technique, internal control loops output current and external control loops output voltage are regulated by PI controllers. In this paper, the Ziegler-Nichols tuning method is used for regulating all the PI controllers [37]. The control technique of the load side VSI is depicted in Fig 15.

Based on the switching frequency of the inverter, the unwanted high frequency harmonics will generate in output ac voltage by the VSI, which ultimately creates power quality problem in the consumer end. Space vector PWM (SV-PWM) technique is used in this controller, because it of slightly reduces the harmonics contents in the output voltage. And also, it increase the fundamental output voltage. To reduce the unwanted high frequency harmonics for avoiding the power quality problem in the customer end, a simple passive L-C filter is used in the load side end. The passive L-C filter design [38] is given in S2 Appendix and the values are:

$$\begin{array}{l} L_{f}=0.052\;H\\ C_{f}=2\mu F \end{array}$$

## Site Selection

**Fig 16:**
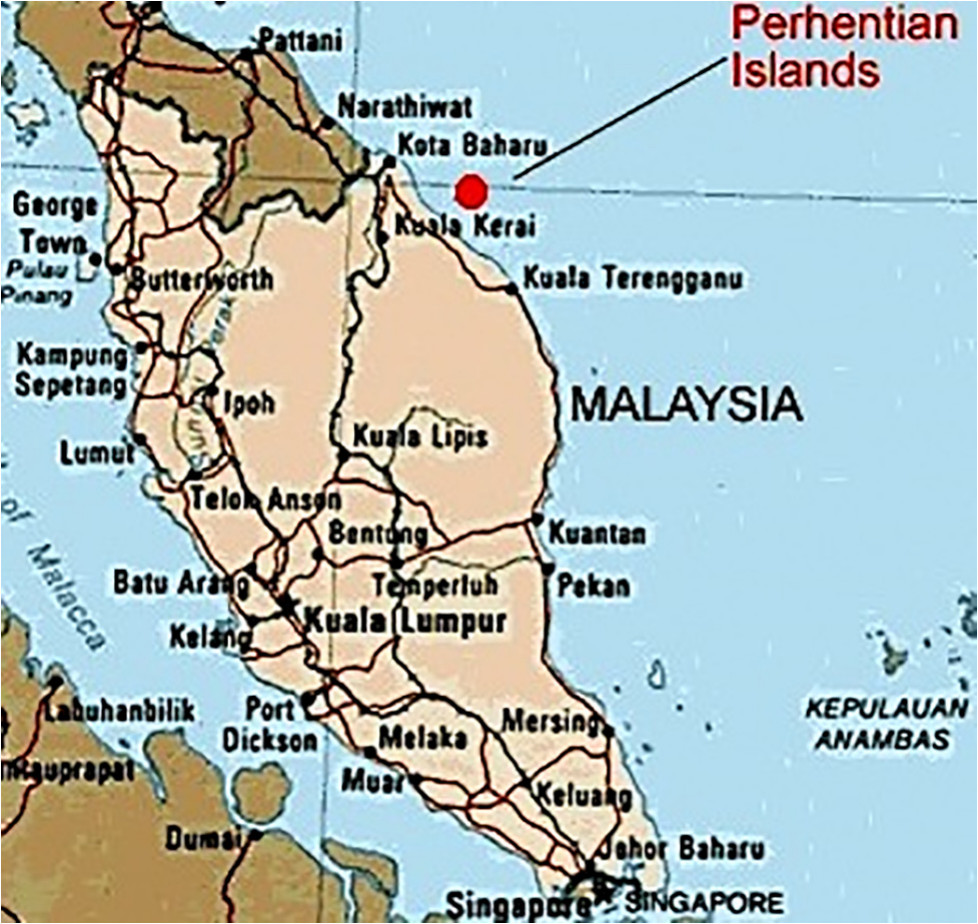
Target site (Perhentian Island) location [41].

Malaysia is surrounded by sea and its latitude and longitude is 2^0^−30′ N and 112^0^–30′E.It has a total of 878 islands[39–40]. In Malaysia, Perhentian Island (shown in Fig 16) is one of the most popular islands resort. It is approximately 20 km off the northeastern coast of West Malaysia in the state of Terengganu. It is a resort island and there is only one village inhabited by the locals. Diesel generators are the main source of electric power. On this island, the National Energy Policies (NEP) and University Kebangsaan Malaysia (UKM) installed a solar-wind hybrid energy system in 2007 [10]. It was not connected to the electrical network because of its weak hybrid power management strategy during periods of lower wind and solar irradiation conditions.

**Fig 17:**
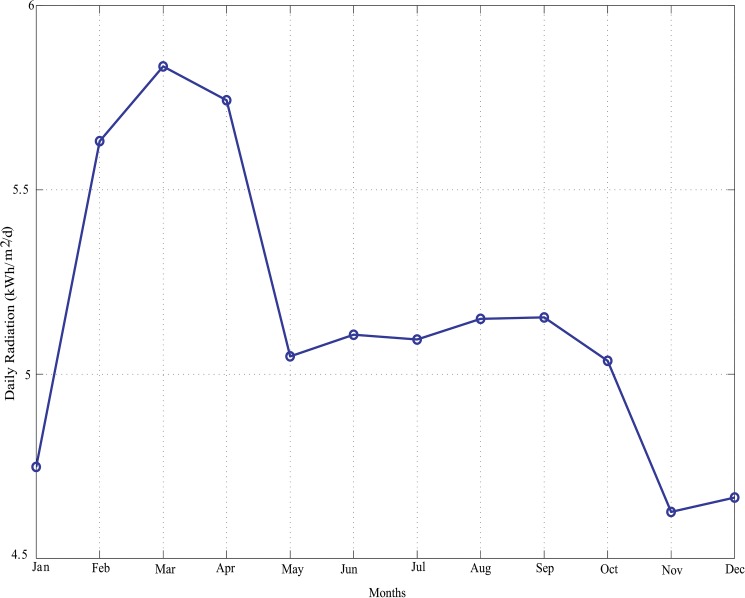
Average monthly solar irradiation of Perhentian Island.

Geographically, Perhentian Island or PulauPerhentian is a hot and humid island with rain all year round. It is a sunny island the equatorial belt. However, a completely clear or cloudless sky is rare on this tropical island. On average, Perhentian Island receives daily 5.5 hours of sunshine daily. The solar data for this island is shown in Fig 17 below.

**Fig 18:**
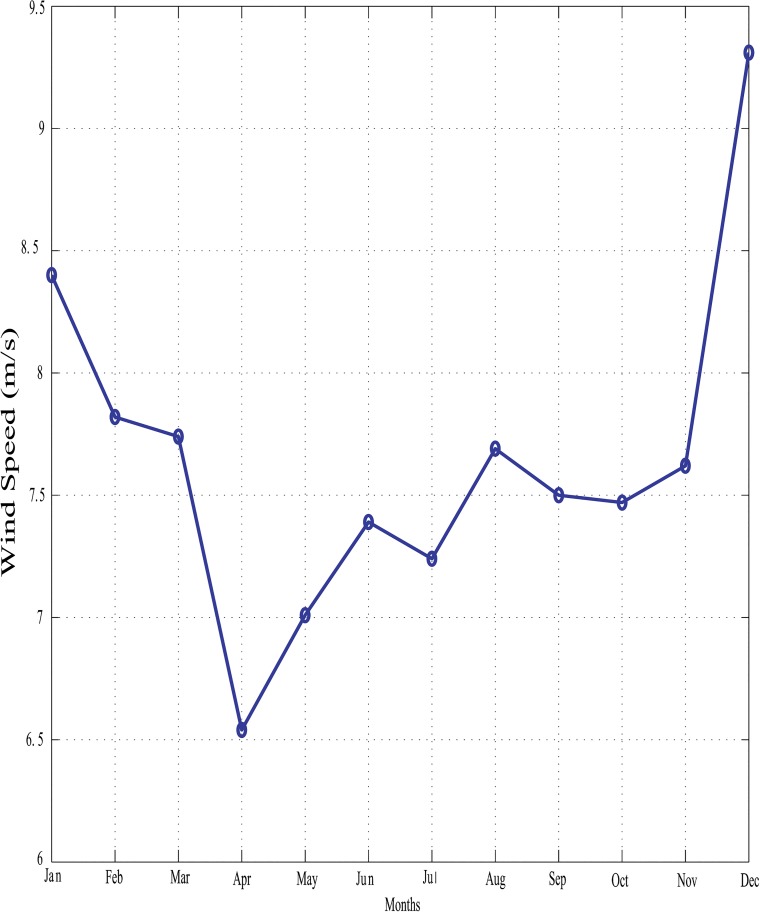
Average monthly wind data for Perhentian Island (2003).

With reference to Fig 16 above, it is clear that solar energy alone cannot meet the customer load demand in every instance because of the limited hours of sunshine. In order to install HRES on this island, wind energy can be considered as it is one of the efficient renewable power generation sources. The wind data for the Perhentain Island is illustrated in Fig 18. Solar and wind data are obtained from references [10] and [42], respectively.

## Simulation Results and Discussion

The simulation model of the proposed standalone solar plus wind energy hybrid system with energy storage has been modelled using a Matlab/Simulink environment under different weather and load conditions. In this study, PMSG is modelled in Matlab/Simulink based on the literature described in [43–44]. The parameters of the PMSG, PV array and wind turbine are taken from the [45], [3, 27] and [46] respectively and are also mentioned in S3 Appendix. In this section, the average solar irradiation for January, February and March; and the average wind speed for January, April, May and June from the section 3 is used to test the performance of the proposed hybrid system under the following conditions.

**Fig 19:**
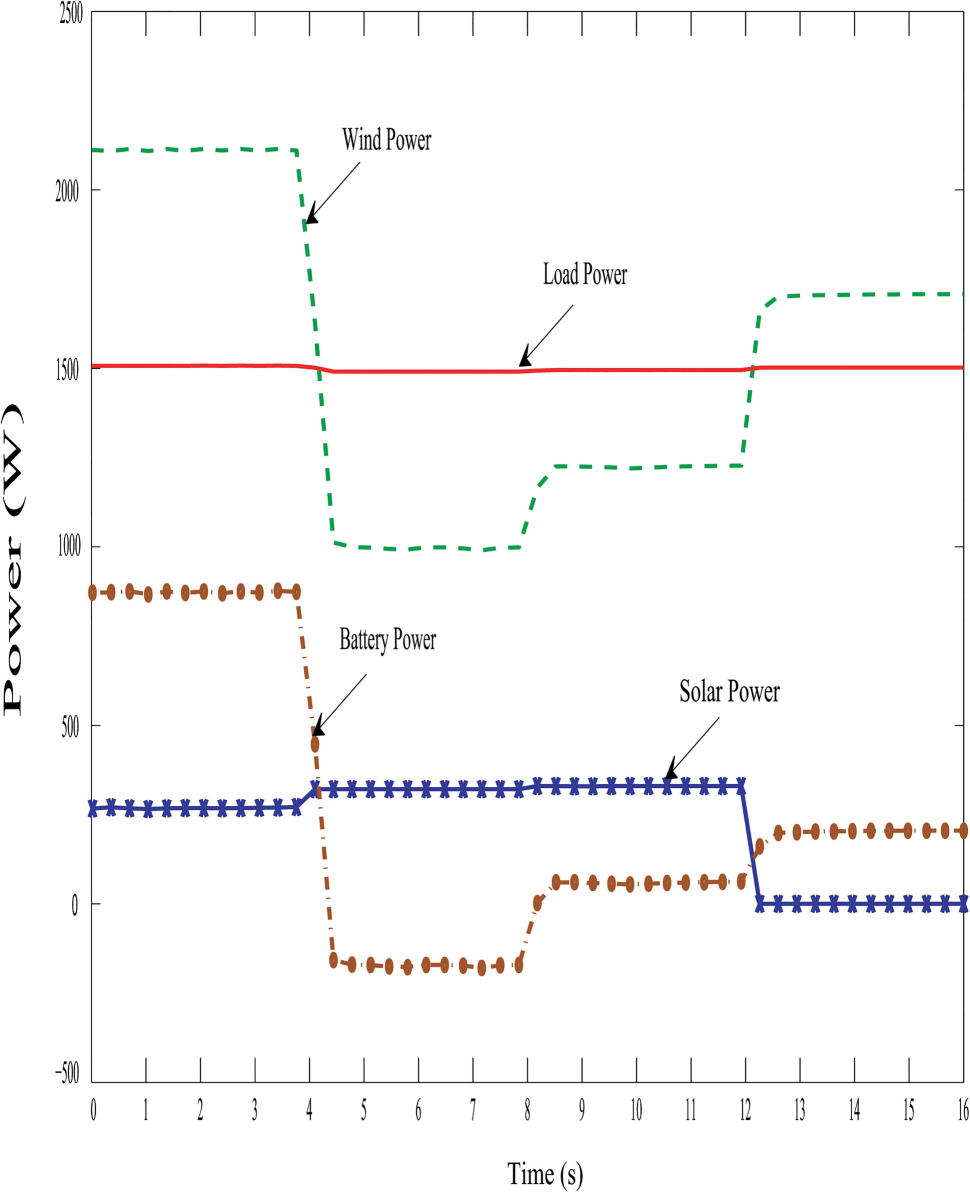
Powers distribution curve of the PV-wind hybrid system during the variation in hybrid power with fixed load.

**Fig 20:**
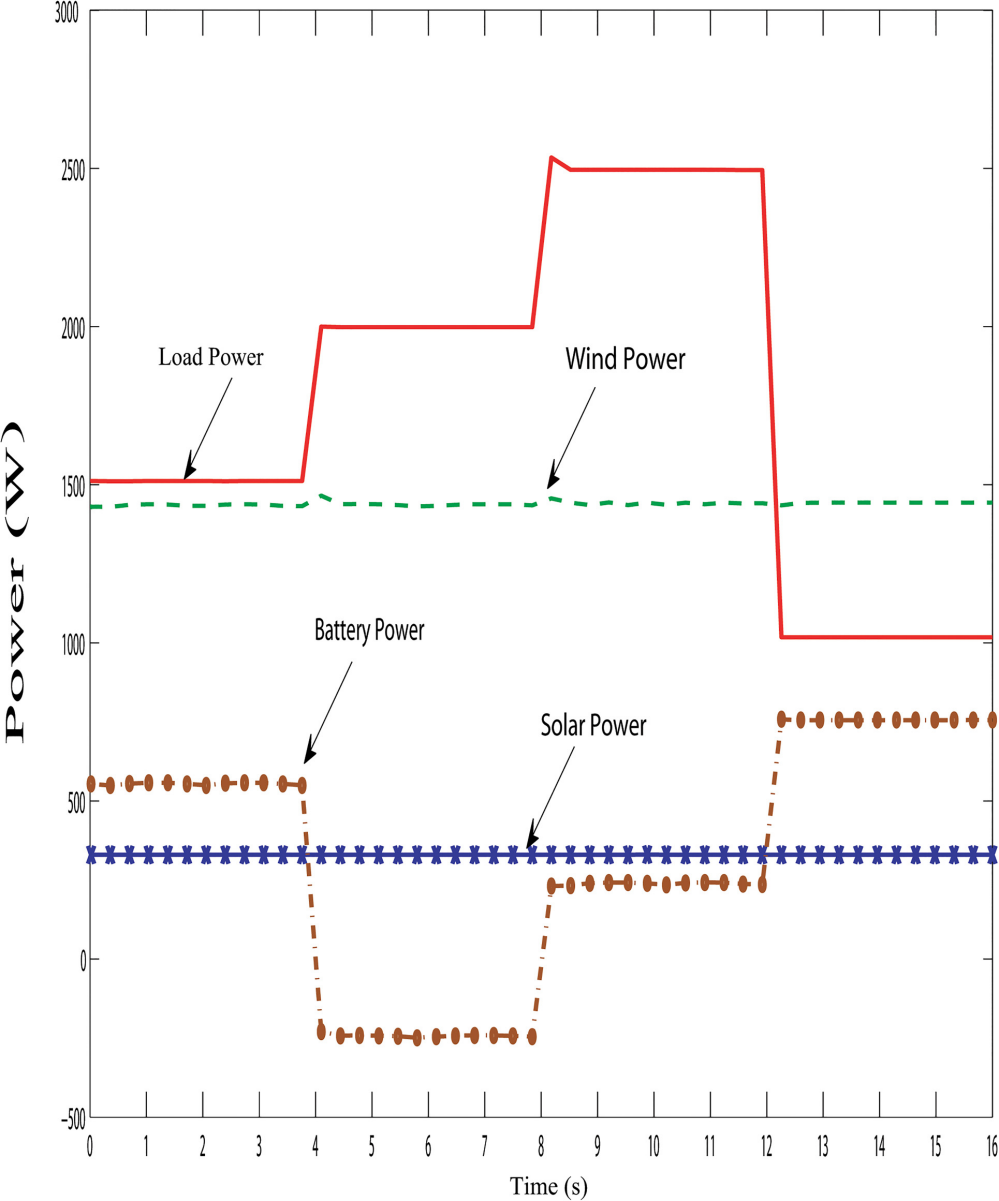
Powers distribution curve of the PV-wind hybrid system during the variation in load with fixed hybrid power.

**Fig 21:**
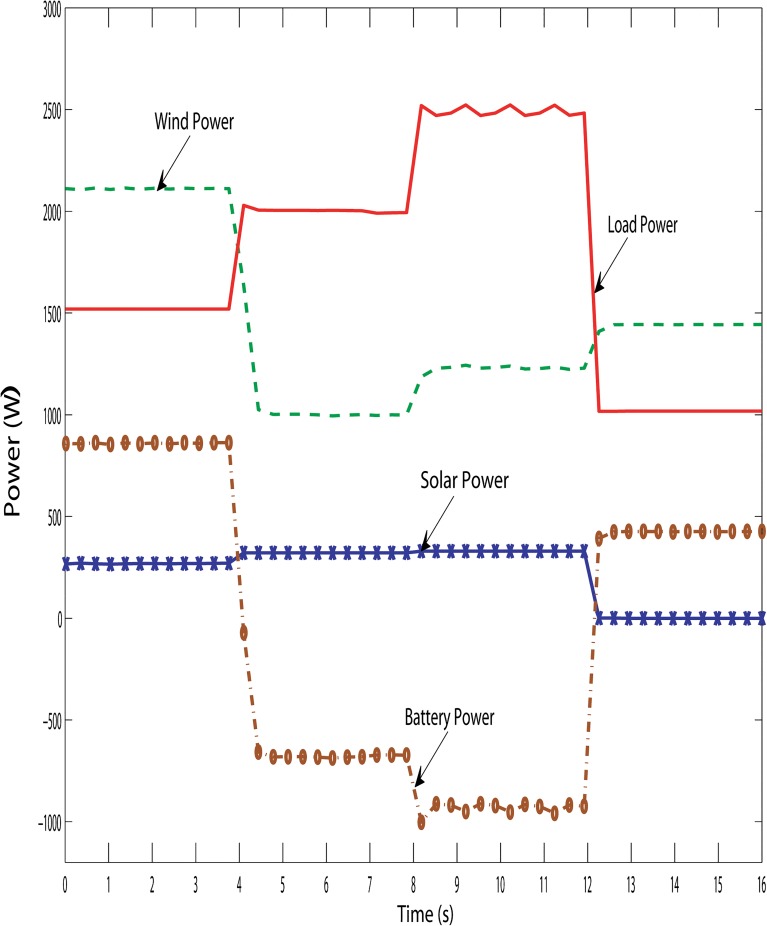
Powers distribution curve of the PV-wind hybrid system during the variation in hybrid power with variable load.

Figs 19, 20 and 21 show the proposed hybrid system power distribution curves under the various conditions: variation in hybrid power with fixed load, variation in load with fixed hybrid power and variation in hybrid power with variable load conditions. From Figs 19, 20 and 21, it is seen that the performance of the DBBC controller is quite satisfactory because the power from the battery bank changes (charges/discharges) in order to maintain system power stability under varying solar, wind and load conditions. So it is evident clear that the DBBC controller is able to discharge the battery bank into the load when the load power is more than the generated hybrid power and also the controller is able to charge the battery bank when the load power is less than the generated hybrid power.

**Fig 22:**
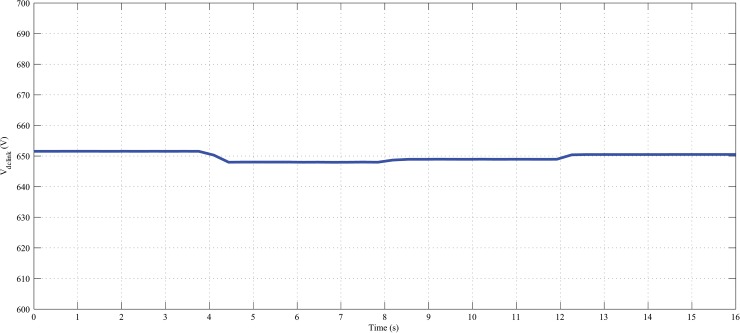
Dc-link voltage of the PV-wind hybrid system during the variation in hybrid power with fixed load.

**Fig 23:**
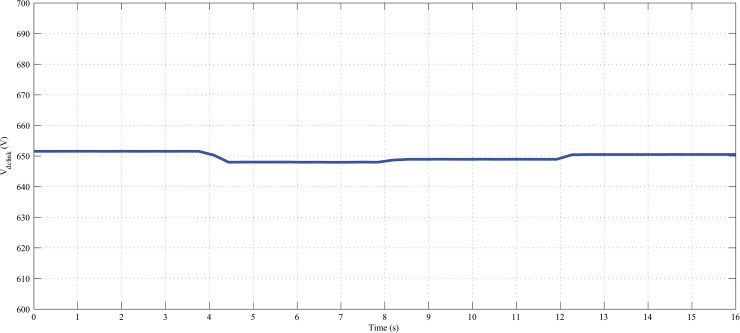
Dc-link voltage of the PV-wind hybrid system during the variation in load with fixed hybrid power.

**Fig 24:**
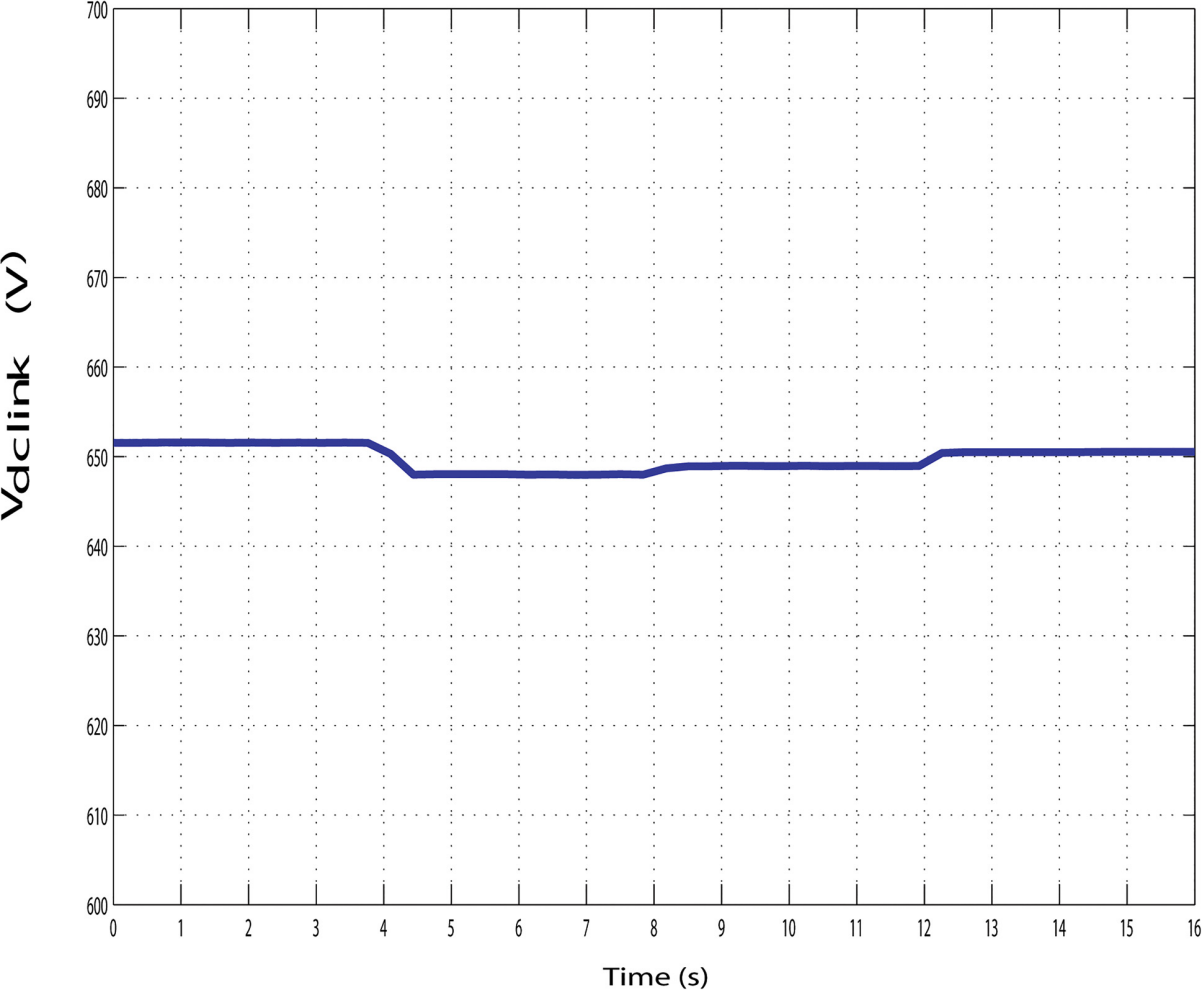
Dc-link voltage of the PV-wind hybrid system during the variation in hybrid power with variable load.

Furthermore, it can also maintained the constant dc-link voltage at 650 V during the change in hybrid and load power, as shown in Figs 22, 23 and 24. The dc-link voltage shown in Figs 22, 23 and 24 are approximately constant but there some insignificant deviation appeared because of the appreciable delay between hybrid and load power changes In Figs 19–21, absence of solar power after 12-second indicate that cloudy and night period. Also in this case, wind and battery bank power satisfactorily meets the system load demand. Therefore, it can be established that the controller performance is quite acceptable in steady state as well as transient hybrid and load power condition.

**Fig 25:**
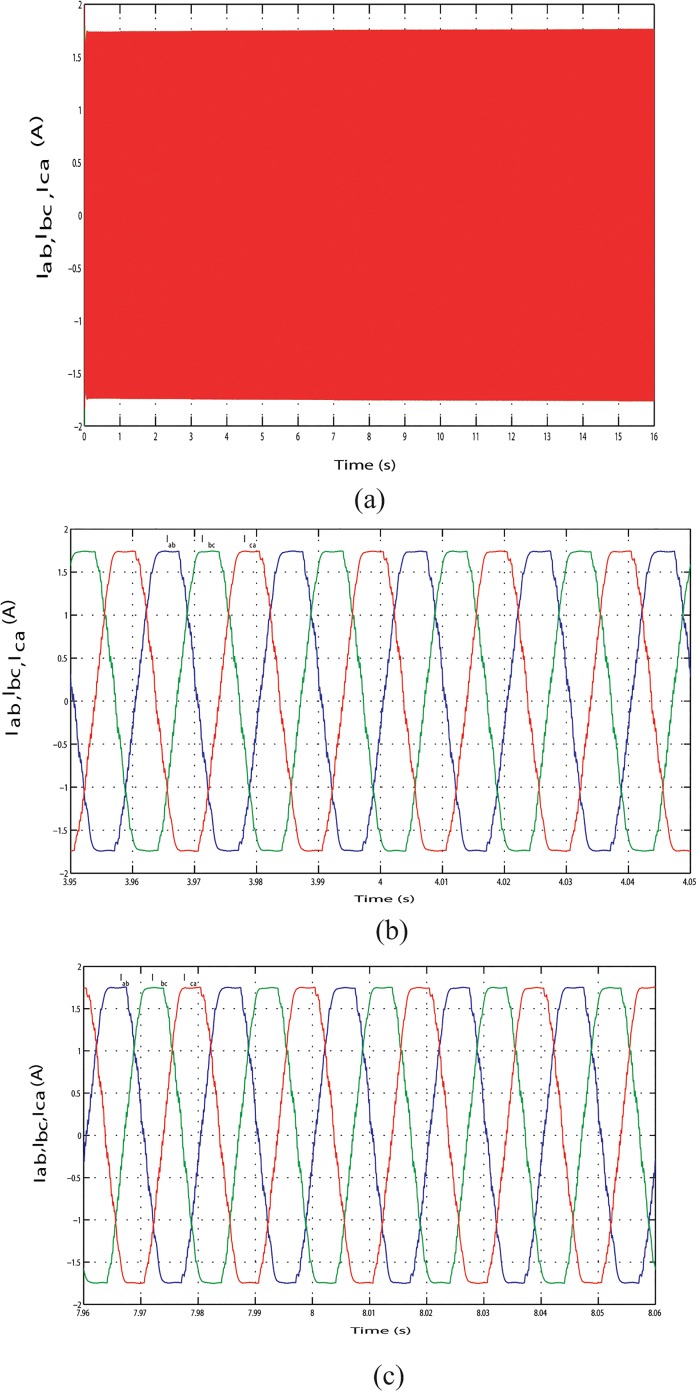
Output line current response with fixed load power. (A) Output line currents throughout the full simulation time. (B) Output line current when the hybrid power decreases at simulation time 3.95 s to 4.05 s.(C) Output line current when the hybrid power increases at simulation time 7.96 s to 8.06 s.

**Fig 26:**
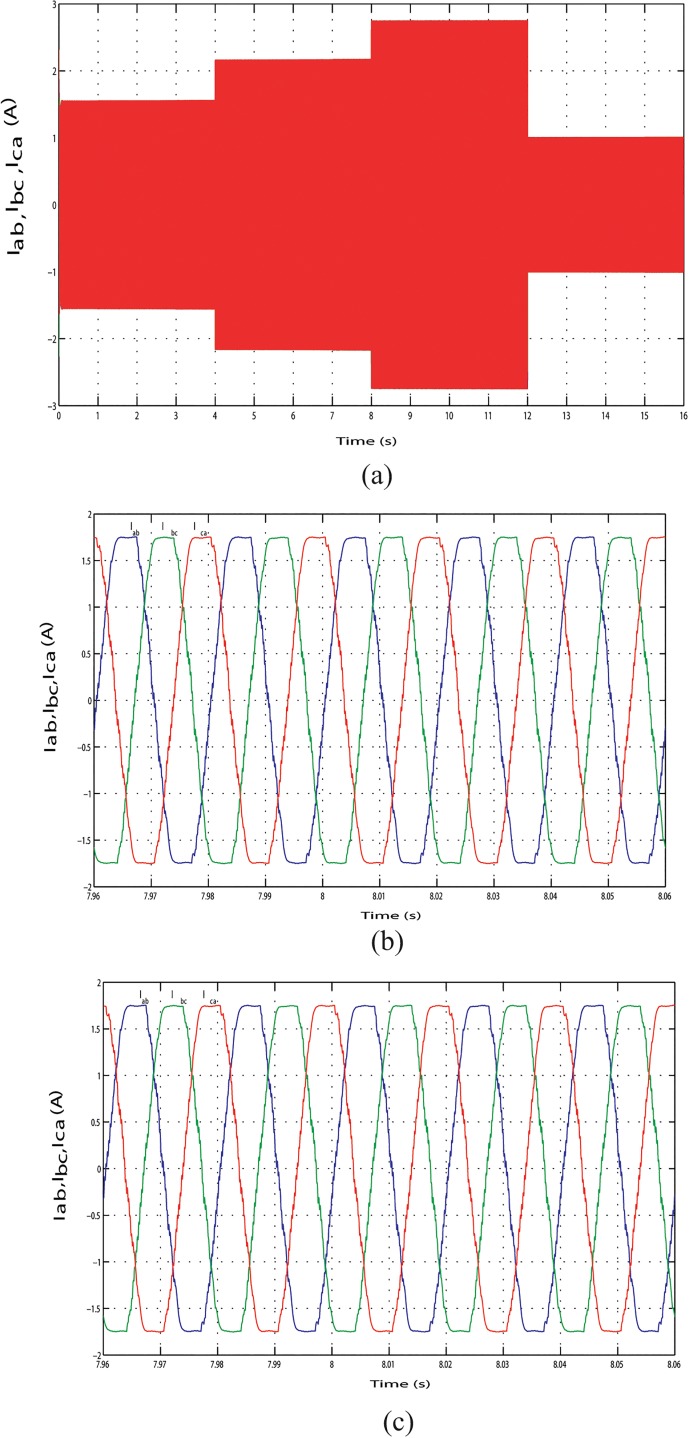
Output line current response with change in required load power. (A) Output line currents throughout the full simulation time. (B) Output line current when the load increases at simulation time 3.96 s to 4.06 s. (C) Output line current when the load decreases at simulation time 11.97 s to 12.07 s.

**Fig 27:**
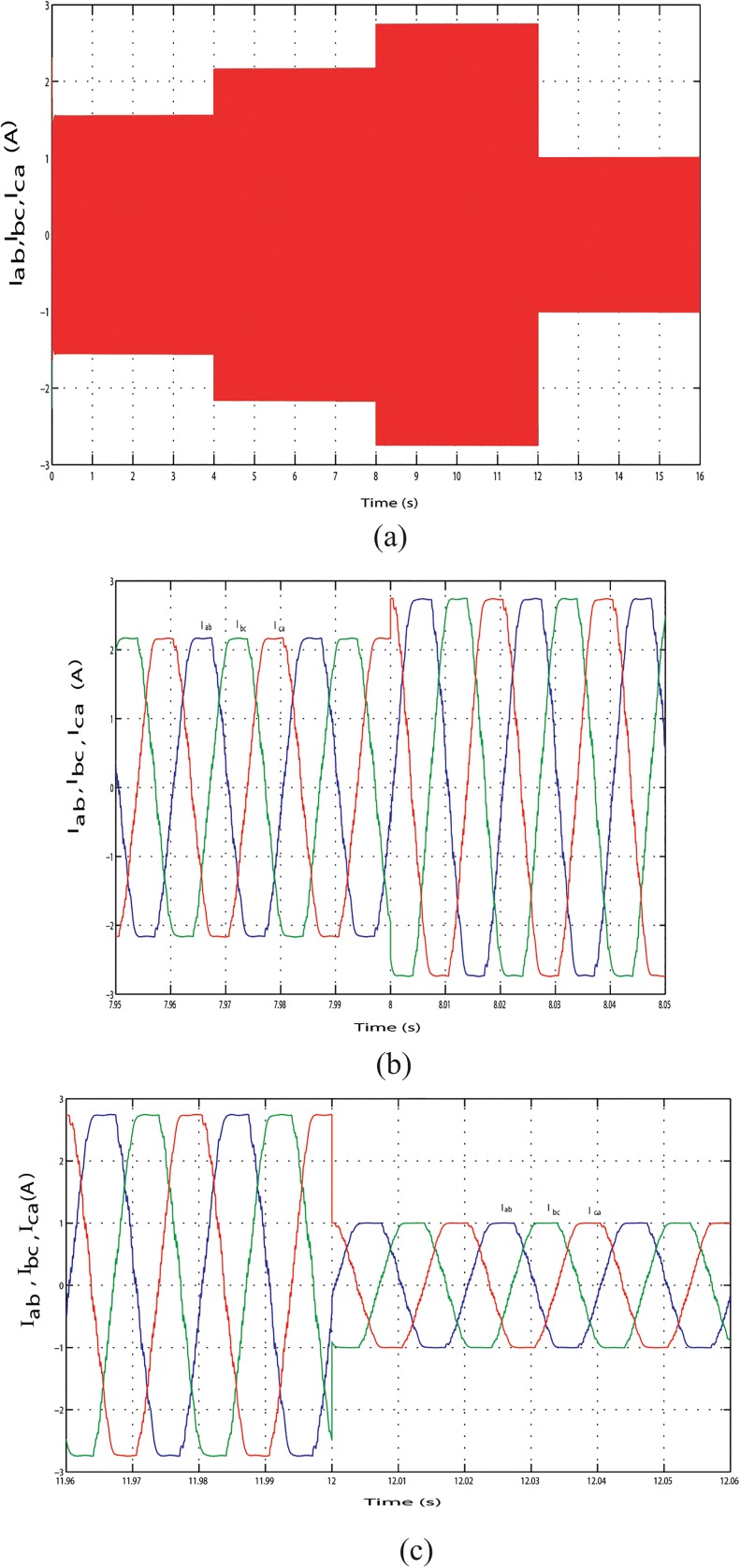
Output line current response with change in required load power. (A) Output line currents throughout the full simulation time. (B) Output line current when the load increases at simulation time 7.95 s to 8.05 s. (C) Output line current when the load decreases at simulation time 11.96 s to 12.06 s.

**Fig 28:**
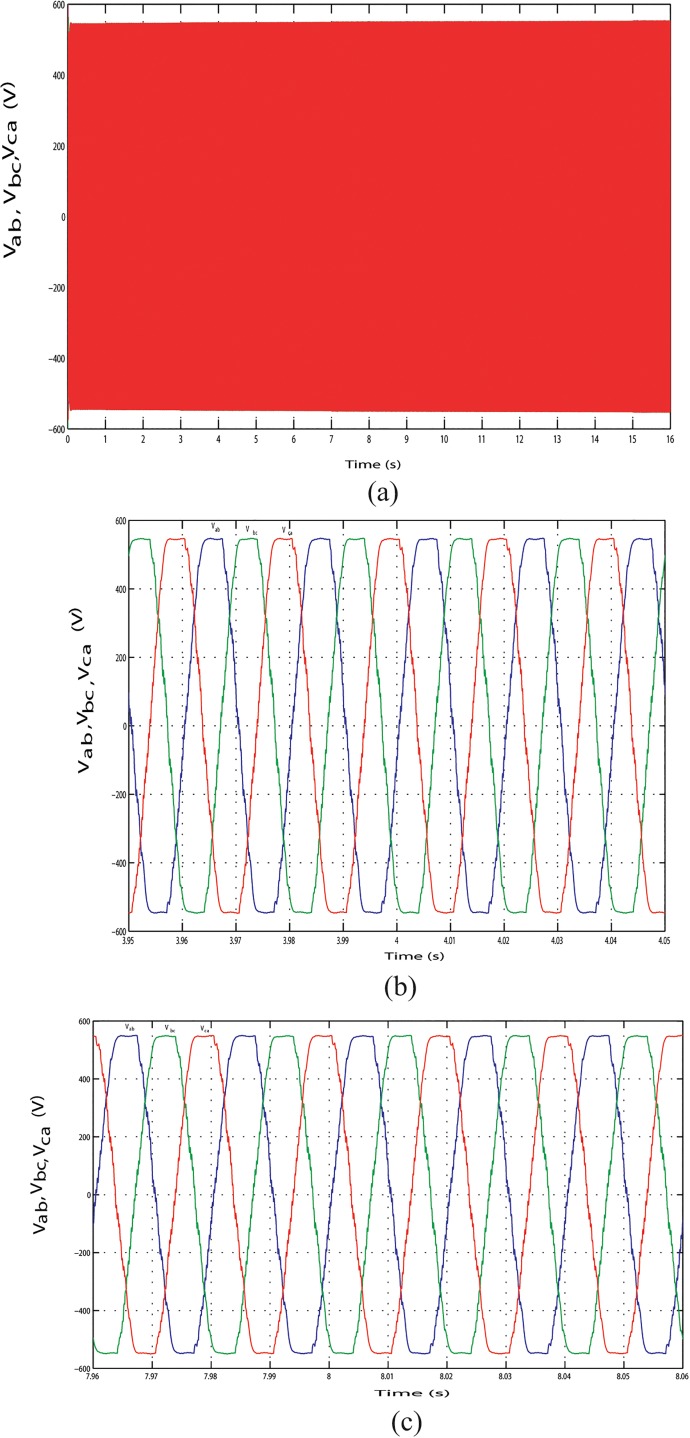
Output line voltage response with fixed load power. (A) Output line voltage throughout the full simulation time.(B) Output line voltage when the hybrid power decreases at simulation time 3.95 s to 4.05 s.(C) Output line voltage when the hybrid power increases at simulation time 7.96 s to 8.06 s.

**Fig 29:**
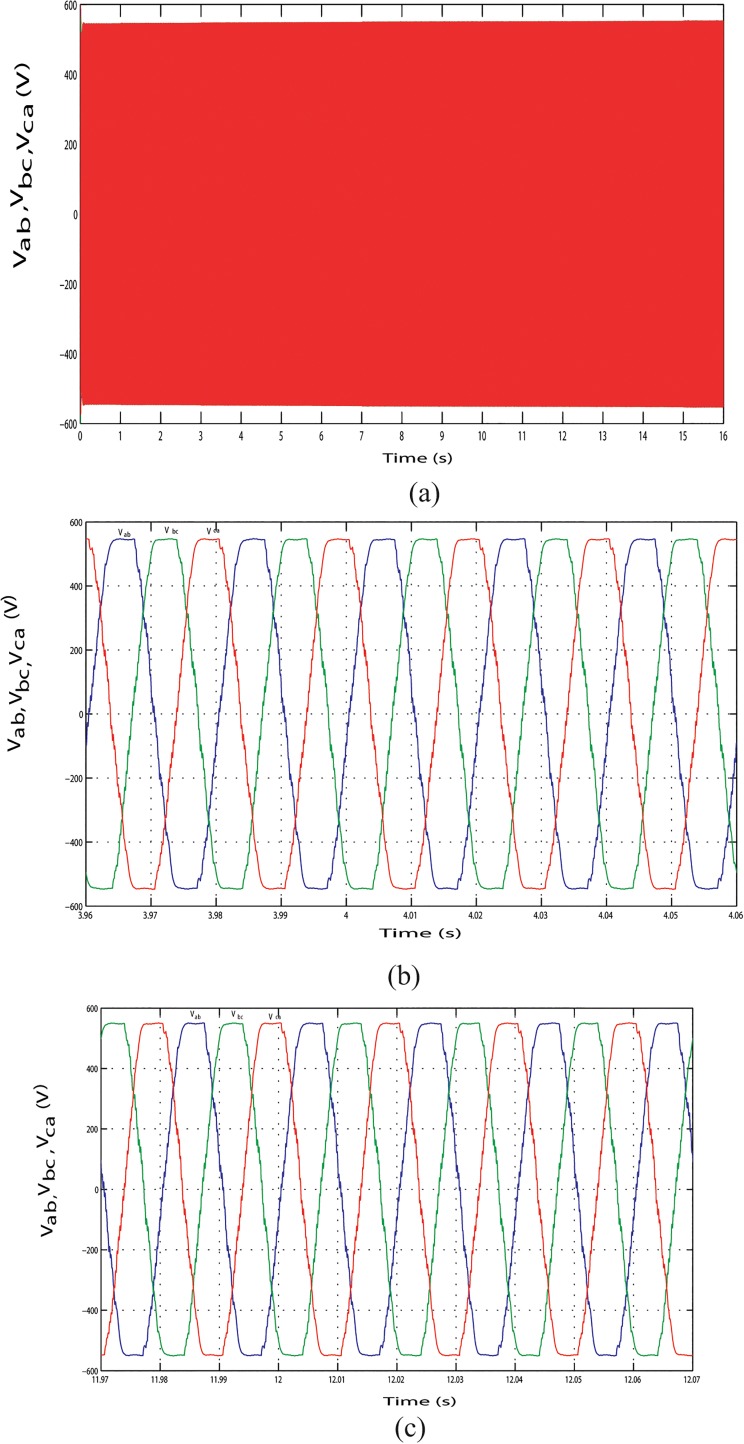
Output line voltage response with change in required load power. (A) Output line voltage throughout the full simulation time. (B) Output line voltage when the load increases at simulation time 3.96 s to 4.06 s;. (C) Output line voltage when the load decreases at simulation time 11.97 s to 12.07 s.

**Fig 30:**
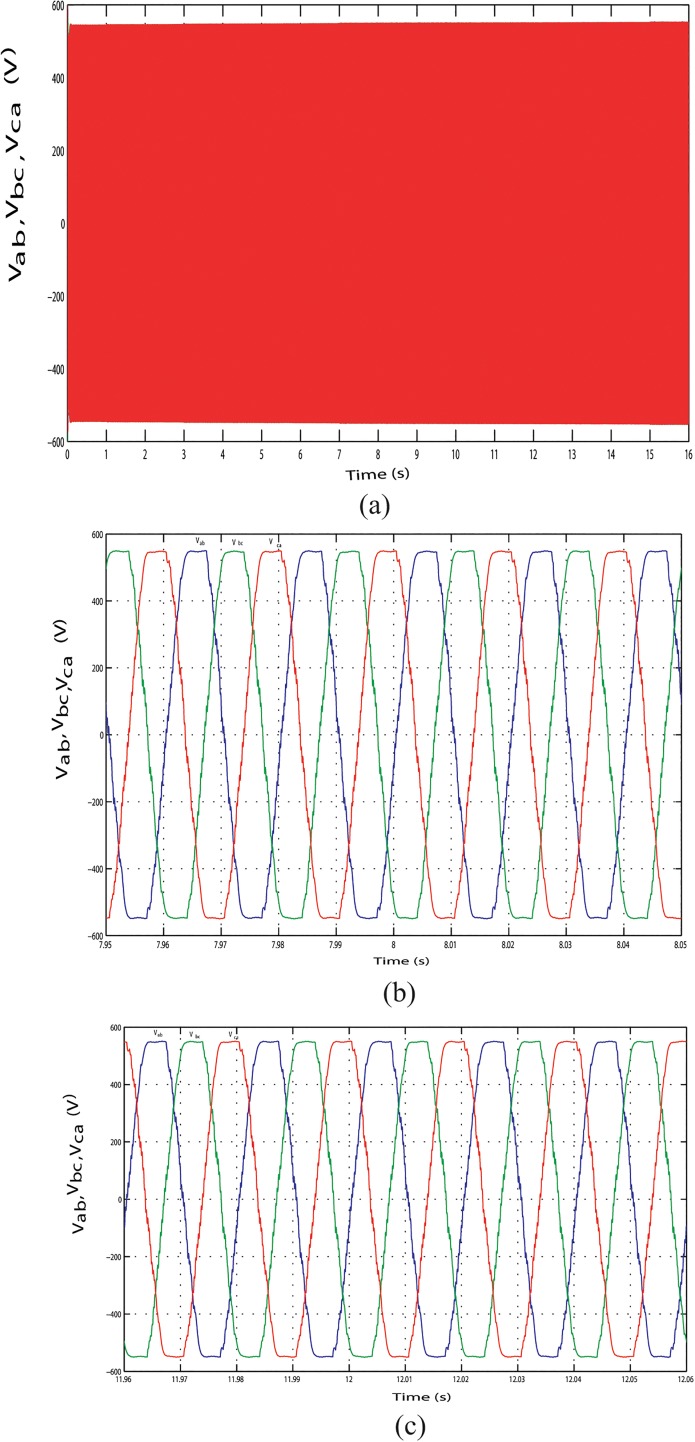
Output line voltage response with change in required load power. (A) Output line voltage throughout the full simulation time. (B) Output line voltage when the load increases at simulation time 7.95 s to 8.05 s. (C) Output line voltage when the load decreases at simulation time 11.96 s to 12.06 s.

The output line current response of the mentioned above three conditions during the long time simulation is shown in Figs 25, 26 and 27. Fig 25(A), 25(B) and 25(C) shows the output line current without variation because load is fixed in this case. The output line current varying in Figs 26 and 27 because load is varying in this case. Fig 26(B) and 26(C) shows the output line current when the load power increase at simulation time 3.96 s to 4.06 s and when the load power decrease at simulation time 11.97 s to 12.07 s, respectively. Similar phenomenon occurred in Fig 27(A), 27(B) and 27(C). For above three conditions, output line voltage for whole simulation time has shown in Figs 28, 29 and 30, respectively. From Figs 28, 29 and 30, it is seen that VSI controller shows satisfactory performance because it maintain voltage stability in load side during the solar, wind and load power variation. The total harmonics distortion in the output voltage and current at load side is about 1.7% and 1.55%, respectively, which illustrates the good quality of voltage and current generated at the load side end.

Based on above simulation results, it can conclude that proposed hybrid system can be able to deliver a suitable quality of voltage and current to the load with the help of inverter switching and a passive L-C filter. Finally, it established that the proposed hybrid system can successfully accommodate solar irradiation, wind speed and load changes, and the power management algorithm can efficiently track the hybrid power and load changes.

## Conclusion

A novel hybrid PV-wind renewable power generation system with appropriate power management algorithm has been designed and modeled in this paper for standalone island uses in the absence of electric power grid. The power available from green energy sources is highly dependent on weather conditions such as solar irradiations and wind speed. In this paper, a PV system integrated with a wind turbine and battery bank using a novel topology to overcome this deficiency. This standalone hybrid topology shows excellent performance under varying load power requirement, solar irradiation and wind speeds where solar irradiation and wind speed data are based on real world records. Based on the simulation results and analysis, it could be concluded that the proposed hybrid system can be satisfactorily used in the Pehentian Islands. Future work should aim at setting up the proposed hybrid standalone PV-wind system in the University of Malaya laboratory to verify the simulation results.

## Supporting Information

S1 Appendix (DOCX)

S2 Appendix (DOCX)

S3 Appendix (DOCX)
