# Supplementary material for: Technical Study of a Standalone Photovoltaic–Wind Energy Based Hybrid Power Supply Systems for Island Electrification in Malaysia
Source: PLoS One. 2015 Jun 29;10(6):e0130678. doi: 10.1371/journal.pone.0130678 (PMC4488286; doi:10.1371/journal.pone.0130678)
Supplement: S1 Appendix — (DOCX) [file pone.0130678.s001.docx]

The battery bank rating calculation:

Battery Rating =$\frac{2.5kW\times1hr}{300V\times0.6}=13.89Ahr$

Hence, a 12-V, 14-Ahr battery rating is considered and consequently, 25 batteries are required to be connected in series.
