# Supplementary material for: Technical Study of a Standalone Photovoltaic–Wind Energy Based Hybrid Power Supply Systems for Island Electrification in Malaysia
Source: PLoS One. 2015 Jun 29;10(6):e0130678. doi: 10.1371/journal.pone.0130678 (PMC4488286; doi:10.1371/journal.pone.0130678)
Supplement: S2 Appendix — (DOCX) [file pone.0130678.s002.docx]

The equations [20] related to passive L-C filter design:

$K=[\frac{k^{2}-\frac{15}{4}k^{4}+\frac{64}{5\pi}k^{5}-\frac{5}{4}k}{1440}^{6}]^{\frac{1}{2}}$ (21)

$L_{f}=\frac{V_{0}}{I_{0}f_{sw}}\{K\frac{V_{dclink}}{V_{0,av}}[1+4\pi^{2}(\frac{f}{f_{sw}})^{2}K\frac{V_{dclink}}{V_{0,av}}]\}^{\frac{1}{2}}$ (22)

$C_{f}=\frac{V_{dclink}}{L_{f}f_{sw}^{2}V_{0,av}}$ (23)

Where

k (modulation index) =1

V_0_ (Load Voltage) =220 V

I_0_ (Load Current) =4.58A

f (fundamental frequency) =50HZ

f_sw_ (switching frequency) =2kHZ

V_o,av_ (total harmonic load voltage) =5% of V_0_

L_f_ Inductance of filter

C_f_ Capacitance of filter
