# Supplementary material for: Technical Study of a Standalone Photovoltaic–Wind Energy Based Hybrid Power Supply Systems for Island Electrification in Malaysia
Source: PLoS One. 2015 Jun 29;10(6):e0130678. doi: 10.1371/journal.pone.0130678 (PMC4488286; doi:10.1371/journal.pone.0130678)
Supplement: S3 Appendix — (DOCX) [file pone.0130678.s003.docx]

**Appendix III:**

**Table 1. Parameters of PMSG**

| Number of poles | 4 |
| --- | --- |
| Rated power | 3 kW |
| Rated speed | 241 rad/s |
| Per phase stator resistance (R) | 0.4578 Ω |
| d-axis and q-axis stator inductance (L_d_ & L_q_) | 0.00334 H |
| Magnetic flux induced in the stator windings (ѱ) | 0.171 Wb |
| Rated torque | 14.2 Nm |

**Table 2. Parameters of Wind turbine**

| Swept area by balde (A) | 12.6 m^2^ |
| --- | --- |
| Air density | 1.22 Kg/m^3^ |
| Rated power | 3000W |
| Diameter of the rotor | 4m |
| Cut in speed | 3 m/s |

**Table 3. Parameters of PV array**

| Maximum rated power (P_max_) | 87 W |
| --- | --- |
| Maximum voltage (V_max_) | 17.4 V |
| Maximum current (I_max_) | 5.02 A |
| Open Circuit voltage (V_oc_) | 21.7 V |
| Short circuit voltage (I_sc_) | 5.34 A |
| Number of Module required | 5 |
